# RUNX2 drives adenoma-to-carcinoma transition in colon cancer

**DOI:** 10.1038/s41419-026-08801-2

**Published:** 2026-04-29

**Authors:** Jin Wu, Kaiyu Shen, Qi Tang, Fan Dong, Zhouyue Gu, Junhao Wu, Xijin Wu, Jiayi He, Zhenhui Zhang, Jie Liu, Bo Fu, Jieya Chen, Yingzi Hu, Ping Ling, Yanxuan Hu, Yu Song, Xia Liu, Morong Wu, Huiying Fu, Yingchao Liu

**Affiliations:** 1 The Second School of Clinical Medicine, Zhejiang Chinese Medical University, Hangzhou, China; 2 The Second Affiliated Hospital of Zhejiang Chinese Medical University, Hangzhou, China; 3 The School of Public Health, Zhejiang Chinese Medical University, Hangzhou, Zhejiang China

**Keywords:** Tumour immunology, Colorectal cancer

## Abstract

Colon adenocarcinoma (COAD), the most common subtype of colon cancer, often arises from adenomas. However, the mechanisms driving the adenoma-to-adenocarcinoma transition remain unclear, hindering early intervention and treatment. Single-cell RNA sequencing revealed that CD8^+^ exhausted T cells (Tex) were significantly enriched in adenoma and carcinoma tissues compared to adjacent normal tissues. Pseudotime analysis and cell-cell communication analysis together revealed a close association between CD8^+^ Tex cells and epithelial cells (EPCs) during the adenoma-to-adenocarcinoma transition. Ligand-target gene interaction and clustering analysis identified the most critical gene set influenced by CD8^+^ Tex in the adenoma-to-adenocarcinoma transition. Among these, runt-related transcription factor 2 (RUNX2) was validated as a critical risk factor through the nine-gene risk score model, TCGA data, and qRT-PCR, demonstrating its role in driving this transition. The RUNX2-specific inhibitor CADD522 suppressed RUNX2 expression in vitro and inhibited the adenoma-to-adenocarcinoma transition in the AOM/DSS model. RUNX2 overexpression promoted proliferation, invasion, migration, and adenoma-to-adenocarcinoma transition-related markers in HCT116 and HCT15 cells, while its knockdown reversed these effects. The tumor necrosis factor receptor superfamily member 1 A (TNFRSF1A) agonist tumor necrosis factor-alpha (TNF-α) upregulated RUNX2 expression and partially mitigated the effects of RUNX2 knockdown. Conversely, TNFRSF1A inhibitor Atrosab downregulated RUNX2 expression and partially reversed RUNX2 overexpression-induced adenoma-to-adenocarcinoma transition. Multiplex immunofluorescence confirmed a close spatial association between CD8^+^PD-1^+^ Tex cells and RUNX2^+^ EPCs. In summary, CD8^+^ Tex cells may activate RUNX2 in malignant EPCs through TNF-α binding to TNFRSF1A, promoting the adenoma-to-carcinoma transition and contributing to poor prognosis.

## Introduction

Colorectal cancer (CRC) ranks third in global incidence and second in cancer-related mortality [1, 2], with over 150,000 new cases projected in 2025 [3]. Colon adenocarcinoma (COAD), the most common CRC subtype, progresses from normal mucosa through adenoma to carcinoma [4]. Most patients are diagnosed at advanced stages when symptoms emerge [5], limiting surgical efficacy [6]. Moreover, chemotherapy and radiotherapy cause significant side effects, while immunotherapy faces resistance and cost barriers [7]. Therefore, identifying drivers of the adenoma-to-carcinoma transition and elucidating underlying mechanisms are crucial for early intervention and treatment of COAD.

During adenoma-to-carcinoma progression, T cell dysfunction, particularly CD8^+^ T cell exhaustion, critically drives tumor development [4, 8, 9]. While tumor-infiltrating CD8^+^ T cells correlate with improved prognosis [10–12], chronic antigen exposure induces exhausted CD8^+^ T cells (CD8^+^ Tex), characterized by impaired effector functions, limited self-renewal, and upregulated inhibitory receptors including PD-1, TIM-3, and LAG-3 [7, 11, 13]. Previous studies have found that in CRC, exhausted CD8^+^ T cells (CD8^+^ Tex) exhibit significant infiltration, with phenotypes closely associated with tumor malignancy [14] and immune evasion [15]. Reports have further revealed the process of CD8^+^ T cell exhaustion in advanced COAD [16]. Recent studies further indicate that exhausted CD8^+^ T cells not only lose their antitumor capabilities but may also promote tumor progression by secreting specific factors [17]. The CD8^+^ Tex subset with high C-X-C motif chemokine ligand 13 (CXCL13) expression interacts with cancer cells, enhancing their invasiveness and facilitating dissemination beyond lymph nodes [17]. However, the role of CD8^+^ Tex in the transition from adenoma to COAD, as well as the key regulatory factors driving this process, remains unexplored.

Tumor necrosis factor-alpha (TNF-α) exhibits dual functions in tumor immunity. Cytolytic CD8^+^ T cells secrete TNF-α that eliminates some tumor cells by triggering extrinsic apoptotic pathways [18, 19]. Conversely, within the tumor microenvironment, certain malignant cells autonomously produce TNF-α, which acts as an autocrine growth factor through nuclear factor kappa B (NF-κB) pathway activation [20]. Beyond direct effects on cancer cells, TNF-α facilitates angiogenesis, stimulates chemokine release that recruits myeloid-derived suppressor cells, and systemically disrupts metabolic homeostasis leading to cachexia [21]. In the immunotherapy context, TNF-α has been identified as a critical mediator of immune checkpoint inhibitor (ICI)-associated adverse events [22, 23]. Clinical evidence demonstrates that TNF-α blockade effectively manages immune checkpoint blockade (ICB)-induced enterocolitis [22], while preclinical mouse models suggest its potential to augment antitumor immunity [24].

Runt-related transcription factor 2 (RUNX2), a master regulator of osteoblast differentiation, has emerged as an oncogenic driver in multiple solid tumors including CRC [25–29]. RUNX2 promotes cancer cell invasion, metastasis, and stemness by regulating genes involved in epithelial-mesenchymal transition (EMT) and extracellular matrix remodeling [27, 30]. While RUNX2 has been implicated in CRC progression [25–27], the potential crosstalk between CD8^+^ Tex-derived TNF-α and RUNX2 activation in adenoma-to-carcinoma transition remains unexplored.

Building on the aforementioned research background, this study focuses on the potential driving role of CD8^+^ Tex in the transition from colorectal adenoma to adenocarcinoma and the associated molecular mechanisms. We propose that CD8^+^ Tex may promote this transition through the activation of the transcription factor RUNX2 via the TNF-α-tumor necrosis factor receptor superfamily member 1 A (TNFRSF1A) signaling axis. This finding provides insight into the tumor-immune microenvironment’s influence on cancer progression and identifies potential targets for early COAD intervention and treatment.

## Results

### Microenvironmental heterogeneity and key cellular subpopulations during COAD progression

**Fig. 1:**
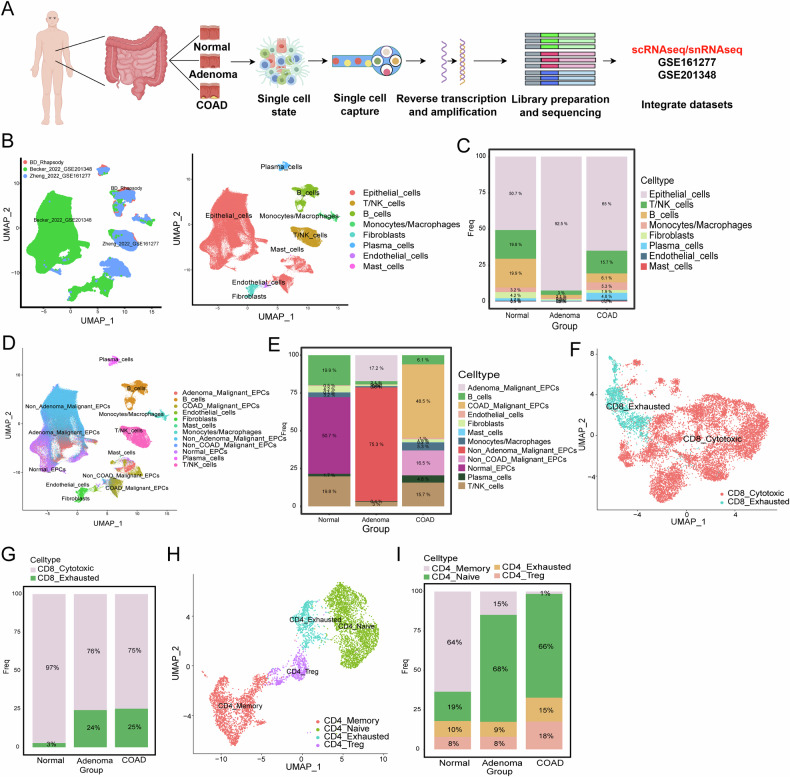
Microenvironmental heterogeneity and key cellular subpopulations during COAD progression. **A** Workflow of sample collection, single-cell sequencing, and data integration. **B** UMAP plot showing the distribution of cells from different datasets and the visualization of major cell types. **C** Bar plot of major cell type proportions in each group. **D** UMAP plot of epithelial cell subtypes and other cell types. **E** Bar plot showing proportions of epithelial subtypes and other cell types across groups. **F** UMAP plot of CD8^+^ T cell subclusters. **G** Bar plot showing the proportions of CD8^+^ cytotoxic and exhausted T cells in each group. **H** UMAP plot of CD4^+^ T cell subclusters. **I** Bar plot showing the proportions of CD4^+^ T cell subtypes in each group.

To comprehensively investigate the evolution of the tumor microenvironment during the adenoma-to-carcinoma progression in colon cancer, we collected colon samples representing three distinct pathological stages: adjacent normal colon tissue (Normal, *n* = 1), adenoma tissue (Adenoma, *n* = 6), and COAD tissue (COAD, *n* = 1). scRNA-seq analysis was performed on these samples using the BD Rhapsody™ platform (Fig. 1A, created by Figdraw, www.figdraw.com). The pathological features of the samples were observed through H-E staining (Fig. S1A). Additionally, we integrated eligible samples from the GSE161277 and GSE201348 datasets, analyzing a total of 12 adjacent normal colon samples, 37 colon adenoma samples, and 8 COAD samples (Fig. 1A).

Following quality control and normalization, 292,770 cells and 36,785 genes were retained for analysis. Harmony [31] was employed to mitigate batch effects in the scRNA-seq data. Cell populations were annotated into eight distinct subtypes—epithelial cells, T/NK cells, B cells, monocytes/macrophages, fibroblasts, plasma cells, endothelial cells, and mast cells—using canonical markers and the CellMarker database. UMAP dimensionality reduction revealed clear spatial segregation and pronounced heterogeneity in cellular composition across pathological stages (Fig. 1B and S1B). Epithelial cells (EPCs) dominated the landscape, followed by T/NK and B cells, while mast cells were the least prevalent. Throughout the progression from normal tissue to adenoma and COAD, EPCs initially expanded but later declined, whereas T/NK cells exhibited an initial reduction followed by an increase, though their proportion remained consistently below that of normal tissue (Fig. 1C).

This study delineated the microenvironmental heterogeneity during the progression from normal colonic mucosa to adenoma and adenocarcinoma. Our findings highlighted substantial diversity within the colonic tumor microecosystem, leading us to concentrate on the detailed characterization and analysis of EPCs and T/NK cells, two pivotal cellular components in tumor progression and immune modulation.

### Identification of malignant EPCs using cancer-finder

Given the central role of malignant cells in tumor progression, we employed the Cancer-Finder algorithm to further distinguish malignant and non-malignant cells in adenoma and COAD samples. Based on their epithelial origins, these cells were re-annotated, and EPCs were subdivided into five subpopulations: COAD malignant EPCs, adenoma malignant EPCs, COAD non-malignant EPCs, adenoma non-malignant EPCs, and normal EPCs. The overall distribution of these subpopulations is shown in Fig. 1D. A gradual increase in malignant EPCs, along with a decrease in non-malignant cells, was observed during the progression from adenoma to COAD (Fig. 1E). This finding highlights the pivotal role of malignant EPCs in driving tumor progression.

### Dynamic remodeling of T cell subpopulations during tumor progression

To explore T/NK cell distribution during the adenoma-to-adenocarcinoma transition, T/NK cells were categorized into CD8^+^ T cells, CD4^+^ T cells, and NK cells, showing distinct clustering patterns in two-dimensional space reflecting unique transcriptomic features (Fig. S1C, D). CD8^+^ T cells were further divided into Exhausted (CD8^+^ Tex) and Cytotoxic (CD8^+^ Tc) subgroups (Fig. 1F and S1E), while CD4^+^ T cells were classified into Memory (CD4^+^ Tmem), Naive (CD4^+^ Tnaive), Exhausted (CD4^+^ Tex), and Regulatory (CD4^+^ Treg) subgroups (Fig. 1H and S1F). Significant changes in immune cell composition were observed, including elevated CD8^+^ Tex proportions in adenoma and COAD tissues (Fig. 1G), suggesting functional suppression of CD8^+^ T cells. CD4^+^ T cell remodeling included reduced CD4^+^ Tmem and increased CD4^+^ Tnaive proportions in adenoma and COAD tissues, with a notable rise in CD4^+^ Treg in COAD tissues (Fig. 1I), likely associated with immune evasion [32]. These findings underscore dynamic alterations in T cell subpopulations, particularly the increase in exhausted and regulatory T cells, contributing to an immunosuppressive tumor microenvironment [33]. Therefore, investigating the roles of T cells within the tumor microenvironment may provide valuable insights into tumor-immune interactions.

### Cell-cell communication analysis highlights the central role of CD8^+^ Tex in the tumor microenvironment

**Fig. 2:**
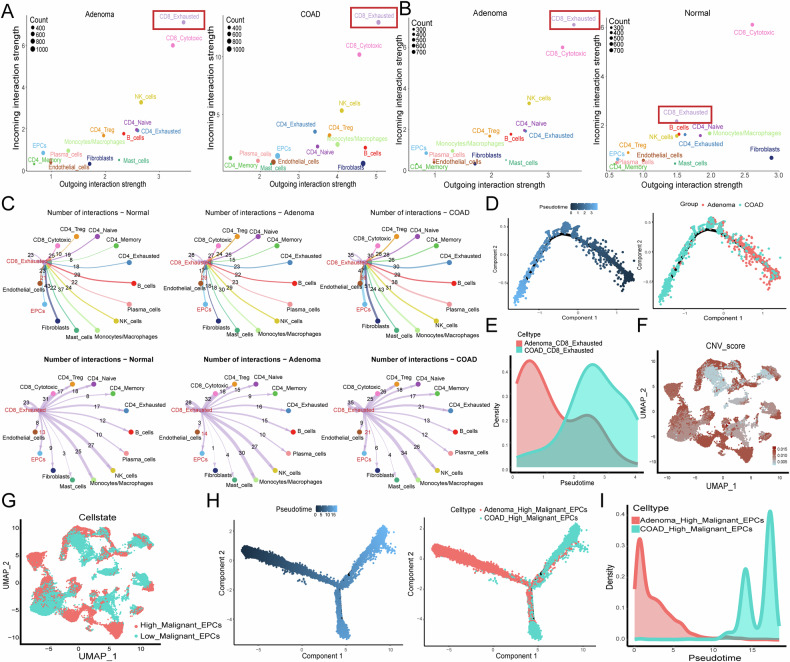
Cell-cell communication and pseudotime analyses reveal the central role of CD8^+^ Tex and their trajectory alongside malignant EPCs in adenoma-carcinoma progression. **A** Scatter plot showing the incoming and outgoing interaction strengths of major cell types in adenoma and COAD tissues. **B** Scatter plot showing the incoming and outgoing interaction strengths of major cell types in normal and adenoma tissues. **C** Circle plot showing the number and strength of interactions between CD8^+^ Tex and all cell populations, including those received and sent. **D** Pseudotime trajectory of CD8^+^ Tex, with their distribution from adenoma and COAD samples along the trajectory. **E** Density plot showing the gene expression density distribution of Adenoma_CD8_Exhausted and COAD_CD8_Exhausted cells along the pseudotime trajectory. **F** UMAP visualization of malignant EPC subpopulations classified by chromosomal CNV score. **G** UMAP plot of malignant EPCs subpopulations based on cell state. **H** Pseudotime trajectory mapping of highly malignant EPCs, with their origin indicated along the trajectory. **I** Density plot showing the gene expression density distribution of Adenoma_High_Malignant_EPCs and COAD_High_Malignant_EPCs along the pseudotime trajectory.

To investigate the communication network features of T cell subpopulations in the tumor microenvironment, we reintegrated T cell subpopulations into their original cellular clusters and applied the CellChat algorithm to visualize intercellular interactions across normal, adenoma, and COAD tissues. The results revealed that CD8^+^ Tex cells exhibited the highest overall communication activity in both adenoma and COAD tissues (Fig. 2A, B). Intriguingly, CD8^+^ Tex cells received the most signal inputs from EPCs, a characteristic that was not prominent in normal tissues. In terms of signal output, CD8^+^ Tex cells demonstrated the strongest overall regulatory influence on other cells within COAD tissues (Fig. 2C).

Pathway information flow analysis revealed distinct interaction patterns of CD8^+^ Tex with other cells across disease stages. In adenoma, CD8^+^ Tex primarily interacted with other cells via pathways such as MK, LCK, MIF, CLEC, CSF, TNF, and IFN-II (Fig. S1G), while in COAD, interactions shifted to CXCL, CEACAM, VCAM, SPP1, and PARs pathways (Fig. S1H). Ligand-receptor analysis indicated that CD8^+^ Tex promoted tumor progression through mechanisms like CD46-JAG1, CD8A-CEACAM5, and TGFB1-TGFBR1/2 (Fig. S2A, B). These findings may highlight CD8^+^ Tex as a key communication hub in the tumor microenvironment and a potential therapeutic target in the adenoma-to-COAD transition.

### CD8^+^ Tex exhibits heterogeneity between adenoma and COAD, accompanied by significant upregulation of CXCL13 during differentiation of adenoma-derived CD8^+^ Tex

Pseudotime analysis of CD8^+^ Tex derived from adenoma and COAD tissues revealed that adenoma-derived CD8^+^ Tex can differentiate into COAD-derived CD8^+^ Tex (Fig. 2D). The gene expression density distribution along the pseudotime trajectory showed that adenoma-derived CD8^+^ Tex cells exhibited higher expression density in the early pseudotime stage, peaking before subsequently declining, indicating increased transcriptional activity during early disease progression. In contrast, COAD-derived CD8^+^ Tex cells exhibited predominant gene expression in the late pseudotime stage (Fig. 2E). These findings support the hypothesis that COAD-CD8^+^ Tex cells may evolve from those in adenoma.

Differential gene expression analysis revealed significant upregulation of CXCL13 during the adenoma-to-COAD transition (Fig. S2C), with pseudotime analysis showing a progressive increase along the differentiation trajectory (Fig. S2D). Given CXCL13^+^ CD8^+^ Tex’s reprogramming effects on tumor cells [17] and their role in regulating tertiary lymphoid structure (TLS) function in CRC [34], this study analyzed interaction networks between CD8^+^ Tex and malignant EPCs to explore their regulatory dynamics during adenoma-carcinoma progression.

### Pseudotime trajectory analysis of highly malignant EPCs

The infercnvpy package was used to calculate chromosomal copy number variations (CNV) scores for malignant EPCs in adenoma and COAD samples (Fig. 2F), classifying them into highly malignant and lowly malignant subpopulations (Fig. 2G). Highly malignant EPCs were further categorized by origin—adenoma-derived or COAD-derived. Subsequently, Monocle2 pseudotime analysis revealed that adenoma-derived highly malignant EPCs could differentiate into COAD-derived highly malignant EPCs, which may include two distinct functional subpopulations (Fig. 2H). Fig. 2I showed that adenoma-derived highly malignant EPCs had higher gene expression density at the early pseudotime stage that subsequently declined, while COAD-derived highly malignant EPCs exhibited predominant expression density at the late pseudotime stage. These findings suggest that highly malignant EPCs in COAD may originate from those present in adenomas. Overall, CD8^+^ Tex cells and EPCs exhibit similar trends in both adenoma and carcinoma, suggesting a potential connection between them.

### Identification of key genes mediating the regulatory effects of CD8^+^ Tex on highly malignant EPCs

**Fig. 3:**
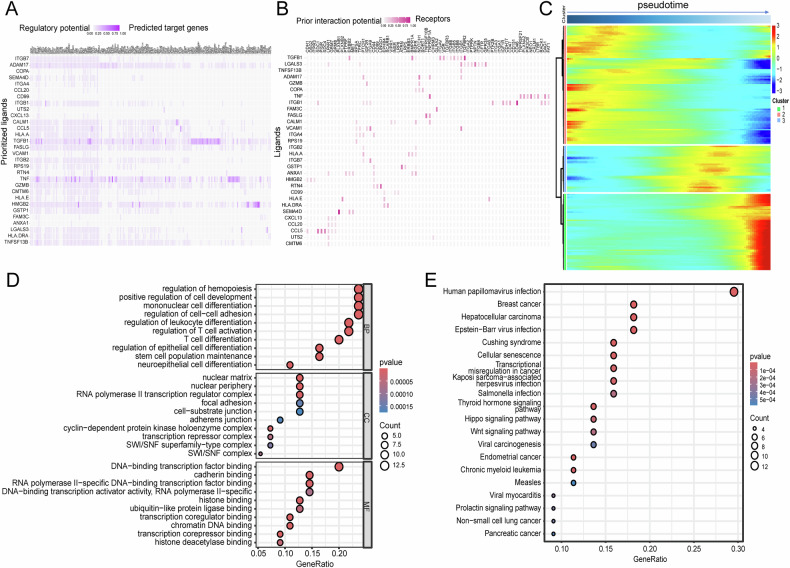
Identification of key genes mediating the regulatory effects of CD8^+^ Tex on highly malignant EPCs. **A** Heatmap showing the top 30 most active ligands expressed by CD8^+^ Tex and their predicted regulatory potential on target genes in highly malignant EPCs. **B** Interaction analysis of dominant ligands in CD8^+^ Tex and their corresponding receptors in highly malignant EPCs. **C** Clustering analysis of target gene expression patterns, classifying genes into three clusters (1-3) along the pseudotime trajectory. **D** GO enrichment analysis of target genes. **E** KEGG enrichment analysis of target genes.

To elucidate the potential regulatory mechanisms of CD8^+^ Tex on highly malignant EPCs, we employed NicheNet to infer potential ligand-receptor interactions and ligand-target gene associations between these two cell populations. As shown in Fig. 3A, the heatmap illustrates the top 30 most active ligands expressed by CD8^+^ Tex and their predicted regulatory potential on target genes in highly malignant EPCs. Notably, the ligands TGF-β1 and TNF in CD8^+^ Tex exhibited the highest regulatory potential on highly malignant EPCs. Further analysis of the interactions between these dominant ligands in CD8^+^ Tex and their corresponding receptors in highly malignant EPCs revealed strong interaction potential for several ligand-receptor pairs, including ITGB1-CD46, SEMA4D-PLXNB1, TGFB1-TGFBR2, and TNF-TNFRSF1A (Fig. 3B).

Clustering analysis of target gene expression patterns in highly malignant EPCs ordered by pseudotime classified them into 3 groups (clusters 1-3, Fig. 3C). As demonstrated in Fig. 2H, this pseudotime progression corresponds to the adenoma-to-COAD transition. Cluster 2 genes showed high expression at early differentiation stages, gradually declining along the pseudotime trajectory. Cluster 3 genes peaked at the intermediate stage, while cluster 1 genes were significantly upregulated during late differentiation. These results suggest that Cluster 1 genes may serve as key drivers in the CD8^+^ Tex-mediated transition of malignant EPCs from adenoma to COAD.

Kyoto Encyclopedia of Genes and Genomes (KEGG) and Gene Ontology (GO) analyses revealed significant involvement of target genes in pathways related to tumor migration, invasion, and differentiation, such as focal adhesion, adherens junction, cadherin binding, and epithelial cell differentiation (Fig. 3D). Besides, tumorigenesis-related pathways, including Wnt and Hippo signaling, were also enriched (Fig. 3E), highlighting mechanisms that drive malignant EPCs from adenoma to COAD through enhanced proliferation and abnormal differentiation.

### Construction of a disease risk prediction model using cluster 1 target genes based on TCGA, GEO bulk RNA-seq, and clinical data

**Fig. 4:**
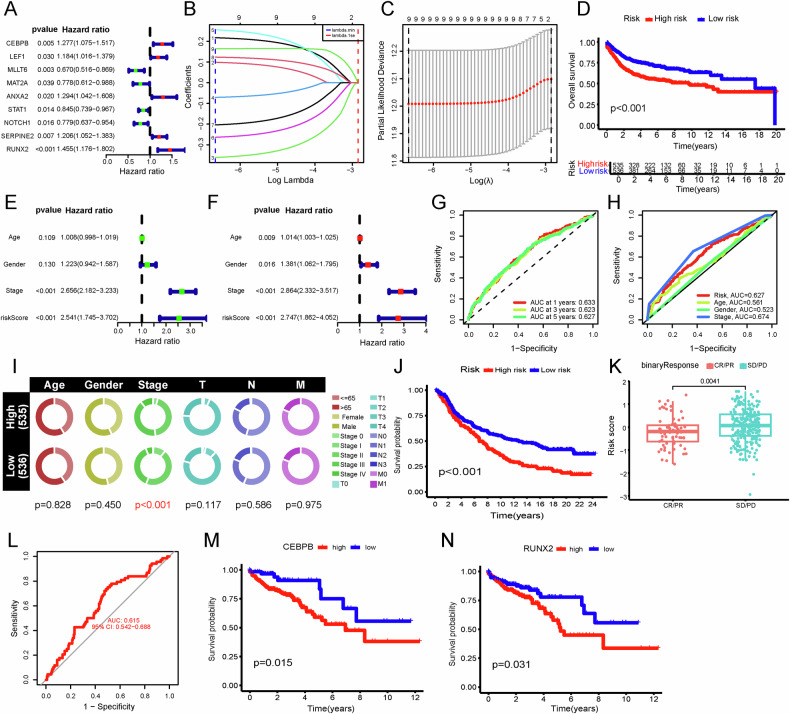
Construction of a disease risk prediction model using cluster 1 target genes. **A** Results of univariate Cox regression analysis for prognosis-related genes. **B**, **C** Feature selection and model construction using LASSO Cox regression. **D** Kaplan-Meier survival analysis comparing high- and low-risk groups. **E** Univariate Cox regression analysis of the relationship between the model and clinical characteristics. **F** Multivariate Cox regression analysis of the relationship between the model and clinical characteristics. **G** Time-dependent ROC curves showing the model’s predictive performance for 1-year, 3-year, and 5-year overall survival. **H** ROC curves showing the predictive performance of risk score, age, gender, and stage for overall survival. **I** Distribution of clinical characteristics in high- and low-risk groups. **J** Kaplan-Meier survival analysis showing survival differences between risk groups in the immunotherapy cohort. **K** Boxplot showing the relationship between disease risk scores and treatment response. **L** ROC curve evaluating the accuracy of the disease risk prediction model in predicting immunotherapy response. **M**, **N** Kaplan-Meier survival analysis of the prognostic significance of individual genes in the TCGA cohort.

Transcriptomic data for 1128 COAD samples from the NCBI GEO database were analyzed. After integration and batch effect correction, expression profiles of cluster 1 genes were extracted and combined with survival information. Univariate Cox regression analysis identified nine prognosis-related genes (*P* < 0.05, Fig. 4A). High expression of CEBPB, LEF1, ANXA2, SERPINE2, and RUNX2 correlated with poor prognosis (hazard ratio [HR] > 1), while MLLT6, MAT2A, STAT1, and NOTCH1 were associated with improved survival outcomes (HR < 1).

To construct a more robust disease risk prediction model, we selected the aforementioned 9 genes and established a Least Absolute Shrinkage and Selection Operator (LASSO) Cox regression model using the glmnet package (Fig. 4B). During lambda selection, the model exhibited optimal performance at the minimum lambda value; therefore, the model containing all 9 genes was selected as the final risk prediction model (Fig. 4C). Risk scores were subsequently calculated for each sample, and samples were stratified into high-risk and low-risk groups based on the median risk score. Survival analysis was then performed, and Kaplan-Meier survival curves were plotted (Fig. 4D). The results demonstrated that patients in the low-risk group had significantly better overall survival (OS) compared to those in the high-risk group (*P* < 0.001). Additionally, survival analysis of each of the nine model genes revealed significant differences, further indicating the stability of the model (Fig. S3A–I).

Univariate and multivariate Cox regression analyses evaluated the association between the risk prediction model and clinical variables, including age, gender, and disease stage (Fig. 4E, F). Intriguingly, while age and gender were not significant in univariate analysis, they emerged as independent prognostic factors in multivariate analysis after adjustment. Both disease stage and the risk model showed significant prognostic value in both analyses, with the model’s predictive ability second only to clinical staging. The performance of the prognostic model was further assessed using a receiver operating characteristic (ROC) curve; an area under the curve (AUC) value closer to 1 indicates stronger predictive ability [35]. All AUC values ranged from 0.5 to 0.7, indicating moderate predictive accuracy (Fig. 4G, H) [36]. Additionally, disease stage significantly differed between high- and low-risk groups, with high-risk patients presenting more advanced disease, consistent with the model’s predictions (Fig. 4I).

Further validation using an immunotherapy cohort (IMvigor210) [37] stratified samples into high- and low-risk groups based on the model formula. Survival analysis revealed that low-risk patients had better prognosis, confirming the model’s predictive value (Fig. 4J, *P* < 0.001). Additionally, patients who responded to immunotherapy exhibited lower risk scores, suggesting that low-risk individuals may benefit more from immunotherapy (Fig. 4K, *P* < 0.05). The ROC curve demonstrated the moderate accuracy of the risk score in predicting immunotherapy response (Fig. 4L).

Survival analysis using the TCGA dataset showed that lower expression of CEBPB and RUNX2 was significantly associated with better prognosis, suggesting their potential as critical prognostic indicators (Fig. 4M, N and Fig. S3J–P). Quantitative real-time polymerase chain reaction (qRT-PCR) analysis of 9 model genes in COAD cell line HCT116 and normal colon epithelial cell line NCM460 revealed consistent trends for RUNX2, CEBPB, MLLT6, MAT2A, and STAT1 with the risk prediction model (Fig. S4A–I). While RUNX2 has been linked to COAD prognosis [26, 27, 38], its role in the adenoma-carcinoma transition remains unclear. Hence, this study aims to clarify RUNX2’s function in this process, providing insights for early intervention and targeted therapy in COAD.

### In vivo and in vitro studies demonstrate that RUNX2 promotes adenoma-carcinoma transition in colon

**Fig. 5:**
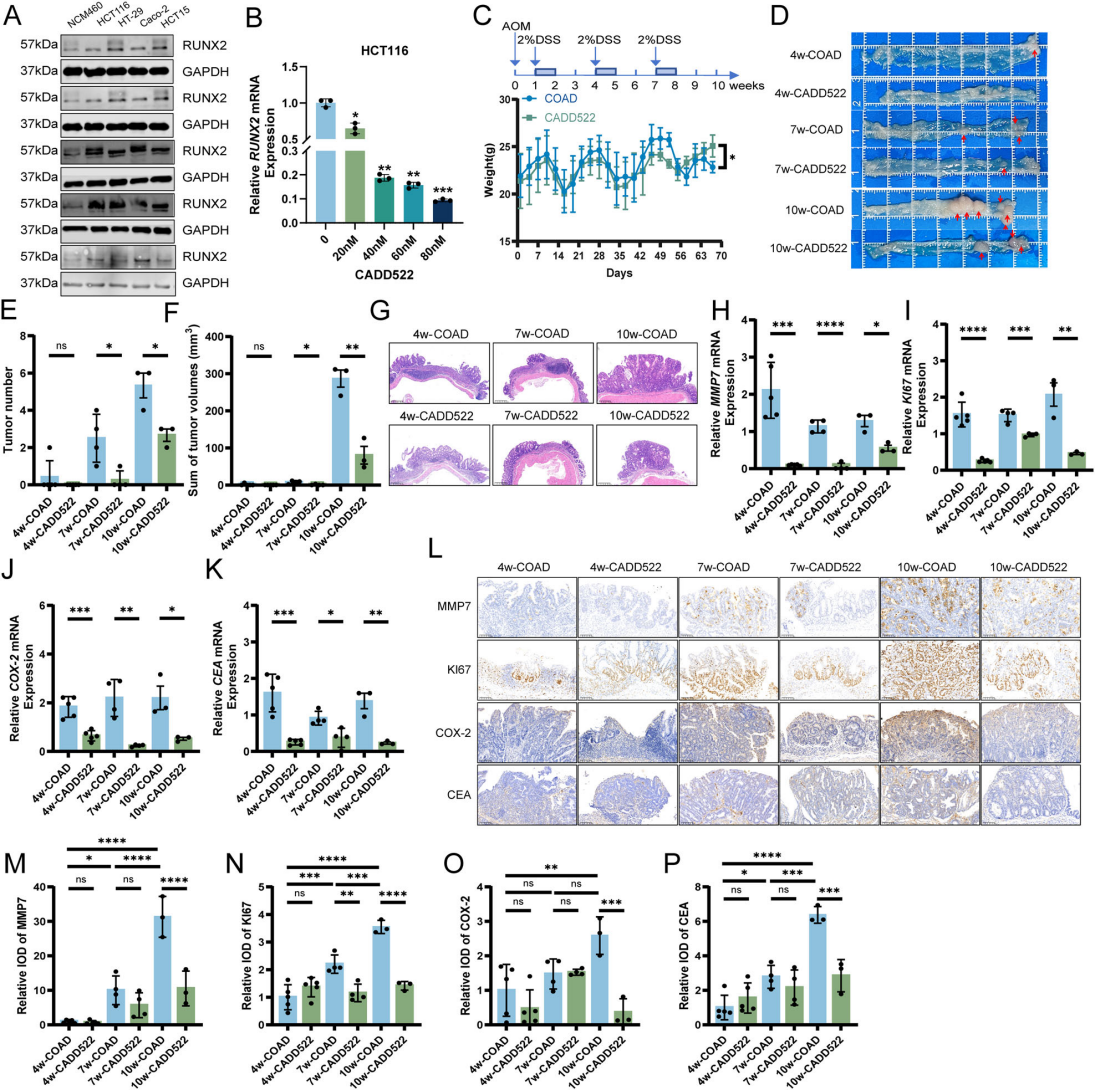
In vivo and in vitro studies demonstrate that RUNX2 promotes malignant progression in COAD. **A** WB analysis showing elevated RUNX2 protein expression in HCT116, HT-29, Caco-2, and HCT15 cells compared with NCM460 cells. **B** *RUNX2* mRNA expression in HCT116 cells treated with different doses of CADD522. **C** Line graph showing body weight changes in mice from the COAD and CADD522 groups. **D** Representative images of colonic tumors in mice from the COAD and CADD522 groups at 4, 7, and 10 weeks. **E**, **F** Quantification of tumor number and volume in each group. **G** Representative H-E staining images of tumors from each group. **H**–**K** Relative mRNA expression levels of *MMP7*, *Ki67*, *COX-2*, and *CEA* in tumor tissues from the COAD and CADD522 groups in vivo. **L** Representative IHC staining of MMP7, Ki67, COX-2, and CEA in tumors from each group. **M**–**P** Bar graphs showing the relative IOD values of MMP7, Ki67, COX-2, and CEA expression in each group.

To elucidate the role of RUNX2 in COAD progression, we conducted Western blotting (WB) analyses, which demonstrated markedly elevated RUNX2 protein levels in HCT116, HCT15, HT-29, and Caco-2 cells relative to normal intestinal epithelial cell line NCM460 (Fig. 5A). To evaluate the inhibitory effect of the RUNX2-specific inhibitor CADD522 on RUNX2 expression and adenoma-to-adenocarcinoma transition, we conducted in vitro and in vivo experiments. In vitro, CADD522 significantly suppressed *RUNX2* mRNA expression in HCT116 cells in a dose-dependent manner, with notable inhibition observed at concentrations of 20-80 nM (Fig. 5B). The downregulation of RUNX2 expression significantly suppressed cell proliferation (Fig. S4J). In vivo, an AOM/DSS-induced CRC mouse model demonstrated that CADD522 treatment significantly reduced tumor number and volume, effectively suppressing the adenoma-to-adenocarcinoma transition, without adversely affecting body weight (Fig. 5C–G).

Further analysis of the expression profiles of key molecular markers involved in the adenoma-to-adenocarcinoma transition (CEA [39], Ki67 [40, 41], p53, MMP7 [42, 43], and COX-2 [44]) revealed that treatment with CADD522 (80 nM) in HCT116 cells reversed the mRNA expression levels of *Ki67*, *MMP7*, and *COX-2* (Fig. S4K–M). Similarly, in the AOM/DSS-induced CRC mouse model, CADD522 intervention led to a marked reduction in *Ki67*, *MMP7*, *COX-2*, and *CEA* mRNA expression at all stages (week 4, week 7, and week 10)—as illustrated in Fig. 5H–K. Importantly, immunohistochemistry (IHC) staining confirmed that CADD522 treatment also significantly suppressed the protein expression of these markers in tumor tissues, with their expression levels showing progressive elevation during tumor progression (Fig. 5L–P), underscoring their pathogenic significance in the adenoma-to-adenocarcinoma sequence.

To better understand its function, we established stable RUNX2-OE and shRUNX2 models in HCT116 and HCT15 cells via lentiviral transduction. The overexpression and knockdown efficiencies were confirmed by qRT-PCR and WB analyses (Fig. S5A–L). In vivo, xenograft models derived from HCT116 cells provided further evidence of RUNX2’s role in tumor progression. Tumors in the RUNX2-OE group exhibited significantly greater volume and weight compared to the Vector-OE group (Fig. S5M, N, Q, R). Conversely, the shRUNX2 group showed a marked tumor-suppressive effect, with significantly reduced tumor volume and weight (Fig. S5O–R).

These findings collectively confirm that RUNX2 promotes the malignant progression of COAD by regulating the molecular network associated with the adenoma-to-adenocarcinoma transition, providing direct experimental evidence for the critical regulatory role of RUNX2 in the development and progression of COAD.

### RUNX2 is a crucial transcription factor in the TNF-α signaling pathway, playing a pivotal role in the adenoma-to-carcinoma transition in COAD

**Fig. 6:**
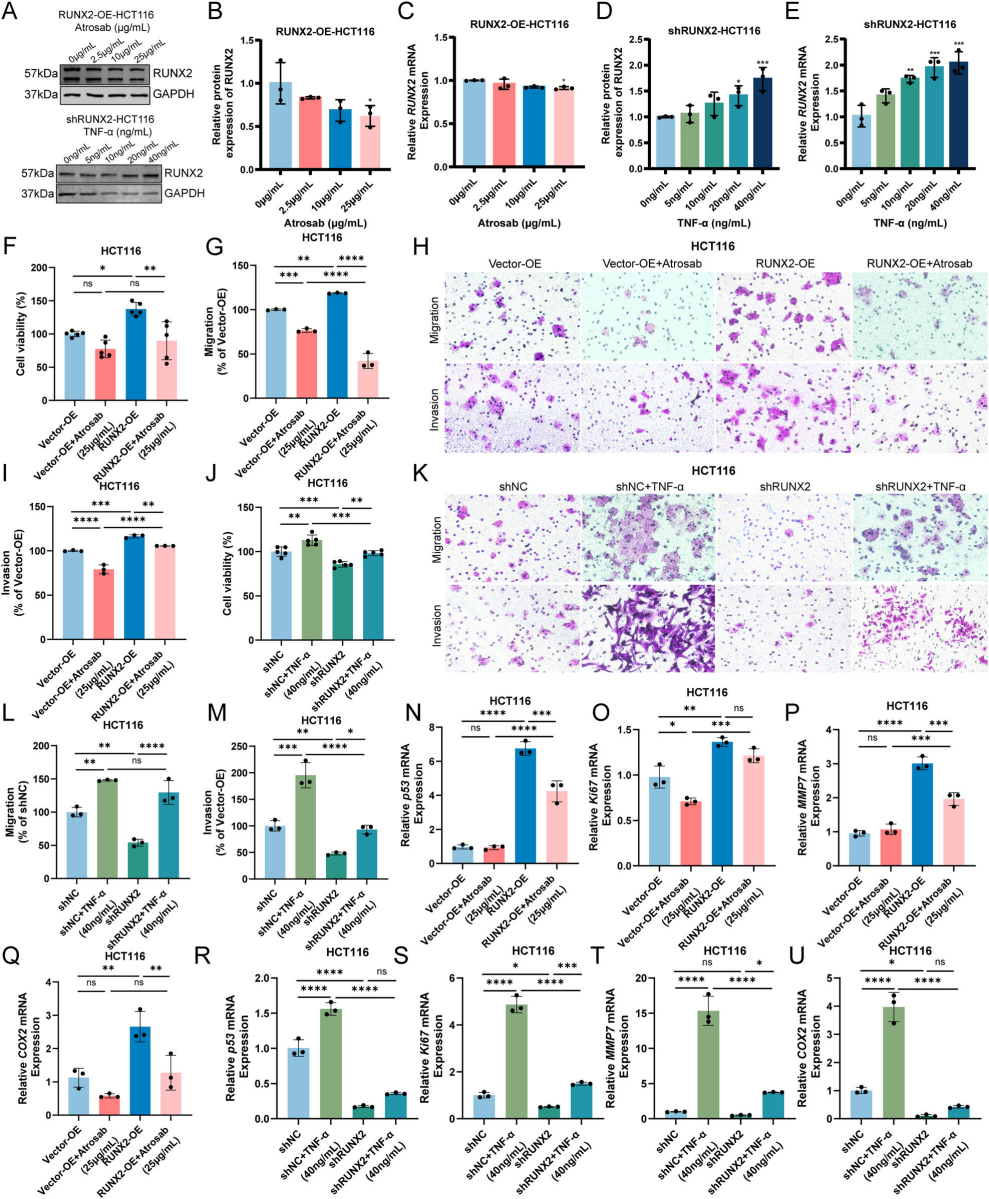
RUNX2 is a crucial transcription factor in the TNF-α signaling pathway, playing a pivotal role in the adenoma-to-carcinoma transition in COAD. **A** RUNX2 protein expression in RUNX2-overexpressing HCT116 cells (RUNX2-OE-HCT116) after treatment with different concentrations of Atrosab and in RUNX2-knockdown HCT116 cells (shRUNX2-HCT116) after treatment with different concentrations of TNF-α, as determined by WB. **B**, **C** Relative RUNX2 protein and mRNA expression in RUNX2-OE-HCT116 cells after treatment with different concentrations of Atrosab. **D**, **E** Relative RUNX2 protein and mRNA expression in shRUNX2-HCT116 cells after treatment with different concentrations of TNF-α. **F**–**I** Effects of the TNFRSF1A inhibitor Atrosab on the proliferation, migration, and invasion abilities of control (Vector-OE) and RUNX2-OE-HCT116 cells. **J**–**M** Effects of the TNFRSF1A agonist TNF-α on the proliferation, migration, and invasion abilities of shNC and shRUNX2-HCT116 cells. **N**–**U** mRNA expression levels of genes associated with adenoma-to-adenocarcinoma transition (*p53*, *Ki67*, *MMP7*, and *COX-2*) in each group of HCT116 cells.

Based on the ligand-target interaction analysis in Fig. 3A and the ligand-receptor interaction analysis in Fig. 3B, TNF derived from CD8^+^ Tex cells and its receptor TNFRSF1A expressed in COAD were identified as a potential upstream signaling axis regulating RUNX2. To comprehensively evaluate the role of RUNX2 in the proliferation, invasion, and migration of COAD cells, as well as its underlying mechanism involving the TNF-α/TNFRSF1A signaling pathway, we conducted functional and intervention assays in HCT116 and HCT15 cells. First, we assessed the association between RUNX2 and the TNF-α/TNFRSF1A pathway using WB and qRT-PCR. Treatment of RUNX2-OE-HCT116 cells with the TNFRSF1A inhibitor Atrosab (0-25 μg/mL) resulted in significant inhibition of RUNX2 protein and mRNA expression at the concentration of 25 μg/mL. Conversely, stimulation of shRUNX2-HCT116 cells with TNFRSF1A agonist recombinant TNF-α protein (0-40 ng/mL) led to a dose-dependent increase in RUNX2 expression, with significant upregulation observed at concentrations of 20 ng/mL and above (Fig. 6A–E). Similarly, using the optimal concentrations (25 μg/mL Atrosab and 40 ng/mL TNF-α), we observed comparable regulatory effects in RUNX2-OE-HCT15 and shRUNX2-HCT15 cells (Fig. S6A–E).

Further functional assays revealed that RUNX2 overexpression significantly promoted cell proliferation, migration, and invasion, whereas RUNX2 knockdown markedly suppressed these cellular behaviors. Mechanistic studies demonstrated that the TNFRSF1A inhibitor Atrosab could only partially reverse the enhancement of proliferation, migration, and invasion induced by RUNX2 overexpression. Similarly, supplementation with the TNFRSF1A agonist recombinant TNF-α protein failed to fully restore the migration and invasion abilities suppressed by RUNX2 knockdown (Fig. 6F–M, Fig. S6F–M).

Subsequently, we assessed the expression of markers (p53, Ki67, MMP7, and COX-2) associated with the adenoma-to-carcinoma transition using qRT-PCR. The results demonstrated that the mRNA levels of these markers were significantly upregulated in the RUNX2-OE group, while RUNX2 knockdown effectively reversed these changes. Intriguingly, the TNFRSF1A inhibitor was unable to effectively suppress the adenoma-to-carcinoma transition-related changes induced by RUNX2 overexpression, and TNF-α supplementation could not fully restore the tumorigenic activity reduced by RUNX2 knockdown (Fig. 6N–U, Fig. S6N–U). Collectively, these findings indicate that RUNX2 may function as an important factor involved in TNF-α signaling pathway that regulates the adenoma-to-carcinoma transition in COAD.

**Fig. 7:**
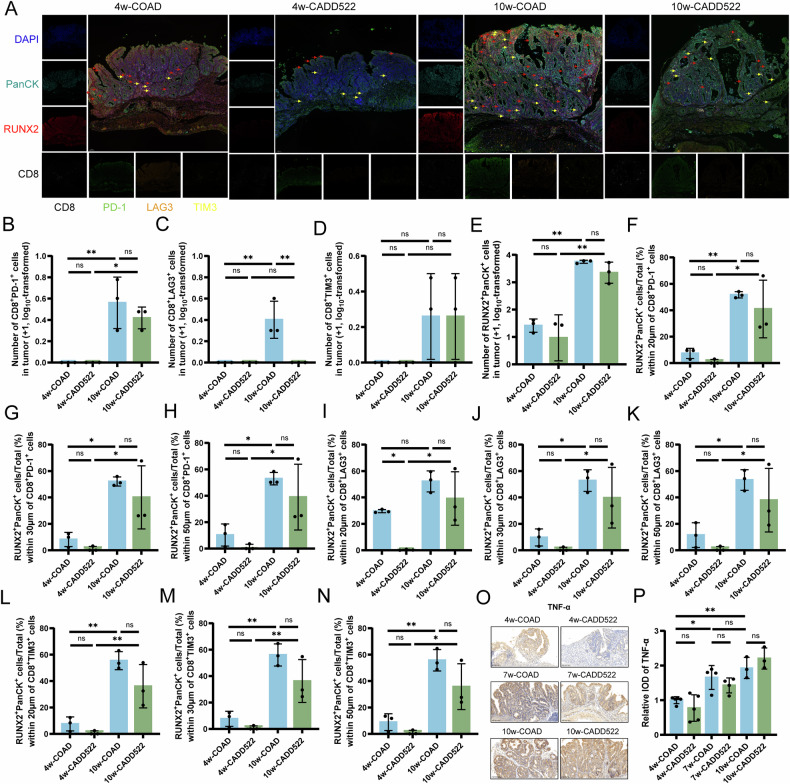
Expression dynamics of RUNX2 during COAD progression and its spatial association with CD8^+^ Tex cells in AOM/DSS-treated mice. **A** Representative mIF images of RUNX2^+^ EPCs (red arrows) and CD8^+^ Tex cells (yellow arrows) in COAD and CADD522-treated groups at 4 and 10 weeks. **B**–**E** Quantification of CD8^+^PD-1^+^ Tex, CD8^+^LAG3^+^ Tex, and CD8^+^TIM3^+^ Tex cells, as well as RUNX2^+^ PanCK^+^ EPCs in COAD and CADD522-treated groups at 4 and 10 weeks. Spatial distance analysis showing the proportion of RUNX2^+^ EPCs within 20 μm (**F**), 30 μm (**G**), and 50 μm (**H**) of CD8^+^PD-1^+^ Tex in each group. Spatial distance analysis showing the proportion of RUNX2^+^ EPCs within 20 μm (**I**), 30 μm (**J**), and 50 μm (**K**) of CD8^+^LAG3^+^ Tex in each group. Spatial distance analysis showing the proportion of RUNX2^+^ EPCs within 20 μm (**L**), 30 μm (**M**), and 50 μm (**N**) of CD8^+^TIM3^+^ Tex in each group. **O** Representative IHC staining of TNF-α in tumors from each group. **P** Bar graph showing the relative IOD values of TNF-α expression in each group.

Additionally, we observed that TNF-α expression levels in colonic tissues progressively increased during COAD development in the AOM/DSS mouse model, and were not significantly reduced by CADD522 treatment (Fig. 7O, P).

### Expression dynamics of RUNX2 in COAD progression and its spatial association with CD8^+^ Tex cells

To investigate the spatial relationship between RUNX2^+^ EPCs and CD8^+^ Tex cells during COAD progression, we performed multiplex immunofluorescence (mIF) staining on colonic tissues from AOM/DSS-treated mice at 4 and 10 weeks using antibodies against RUNX2, PanCK, CD8, PD-1, LAG3, and TIM3 (Fig. 7A). In vehicle-treated COAD mice, the numbers of CD8^+^ PD-1^+^ and CD8^+^ LAG3^+^ Tex cells, as well as RUNX2^+^ PanCK^+^ EPCs, significantly increased from week 4 to week 10 (Fig. 7B–E), consistent with tumor progression. Spatial proximity analysis revealed that the proportion of RUNX2^+^ PanCK^+^ EPCs within 20 μm, 30 μm, and 50 μm radii of CD8^+^ PD-1^+^, CD8^+^ LAG3^+^, and CD8^+^ TIM3^+^ Tex cells progressively increased during tumor development (Fig. 7F–N), suggesting that RUNX2^+^ EPCs become spatially enriched in the CD8^+^ Tex cell microenvironment as tumors progress. Notably, CADD522 treatment did not significantly alter the number of CD8^+^ Tex cells and RUNX2^+^ PanCK^+^ EPCs at week 10 compared with COAD group (Fig. 7A–E). These observations are consistent with the mechanism of action of CADD522, which functions as a transcriptional inhibitor by blocking RUNX2-DNA binding without reducing RUNX2 protein expression or altering upstream inflammatory signals [45] (Fig. 7O, P).

**Fig. 8:**
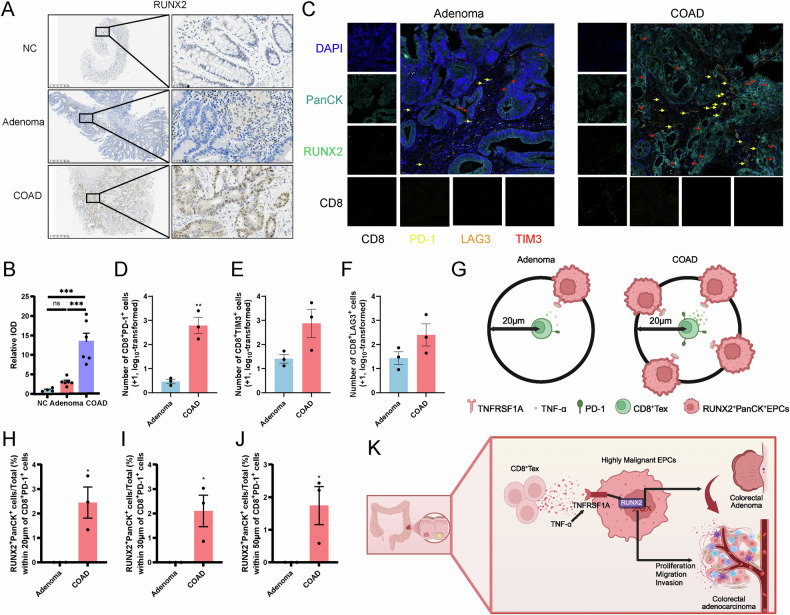
Expression dynamics of RUNX2 during COAD progression and its spatial association with CD8^+^ Tex cells in clinical samples. **A** Representative IHC staining of RUNX2 in adjacent normal tissues (NC), adenoma, and COAD tissues. **B** Bar graph showing the relative IOD values of RUNX2 expression in each group. **C** Representative mIF images of RUNX2^+^ EPCs (red arrows) and CD8^+^ Tex cells (yellow arrows) in human colon adenoma and COAD tissues. **D**–**F** Quantification of CD8^+^PD-1^+^ Tex, CD8^+^LAG3^+^ Tex, and CD8^+^TIM3^+^ Tex cells in adenoma and COAD tissues. **G** Schematic diagram of spatial distance analysis. Created in BioRender. Song, Y. (2026) https://BioRender.com/kq8uqya. Spatial distance analysis showing the proportion of RUNX2^+^ EPCs within 20 μm (**H**), 30 μm (**I**), and 50 μm (**J**) of CD8^+^PD-1^+^ Tex cells in each group. **K** Mechanism diagram of this research. Created in BioRender. Song, Y. (2026) https://BioRender.com/tyqnvdw.

To validate these findings in human COAD, we analyzed tissue sections from colorectal adenomas (*n* = 6), COAD (*n* = 6), and adjacent normal colon (*n* = 4). IHC results showed that RUNX2 expression was significantly elevated in COAD tissues compared to adjacent normal colon and adenoma tissues (*P* < 0.05, Fig. 8A, B). MIF revealed that the number of CD8^+^ PD-1^+^ Tex cells was significantly increased in COAD compared to adenomas (Fig. 8C, D), while CD8^+^ LAG3^+^ and CD8^+^ TIM3^+^ Tex cells showed non-significant trends (Fig. 8E, F). Spatial proximity analysis demonstrated that the proportion of RUNX2^+^ EPCs within 20-50 μm radii of CD8^+^ PD-1^+^ Tex cells was significantly higher in COAD compared to adenomas (Fig. 8G–J), while the spatial association with CD8^+^ LAG3^+^ or CD8^+^ TIM3^+^ Tex cells did not differ significantly (Fig. S7A–F). These findings demonstrate that the progressive spatial enrichment of RUNX2^+^ EPCs near CD8^+^ Tex cells, particularly PD-1^+^ cells, is conserved in human COAD, supporting the clinical relevance of the TNFRSF1A-RUNX2 axis in driving tumor progression.

## Discussion

In this study, we integrated self-generated data with two GEO datasets to perform scRNA-seq analysis on normal, adenoma, and COAD samples. Through prognostic model construction, clinical sample validation, and in vitro and in vivo experimental verification, we revealed the driving role of CD8^+^ Tex cells in the transition of adenomatous malignant EPCs to COAD malignant EPCs. Mechanistically, TNF-α secreted by CD8^+^ Tex cells may activate the TNFRSF1A receptor on EPCs, subsequently upregulating RUNX2 expression and promoting malignant transformation.

Accumulating evidence has established the multifaceted role of CD8^+^ Tex cells in CRC pathogenesis, yet their specific contribution to adenoma-to-adenocarcinoma progression remains unexplored. Previous studies have primarily focused on CD8^+^ Tex cells in established CRC. Xiaogang Shen et al. demonstrated that CD8^+^ Tex cell abundance correlates with prognosis and immunotherapy outcomes [7]. Xi Yang et al. showed extensive crosstalk between tumor-infiltrating CD8^+^ Tex cells and Tregs via CCL4-CCR8 signaling [46]. Zixu Chen’s team identified CXCL13^+^ CD8^+^ Tex cells as regulators of CRC-associated tertiary lymphoid structures [34]. Tianlong Ling’s group revealed that Tex cells interact with EPCs through ADGRE5-CD55 and CD8A-CEACAM5 axes to facilitate CRC liver metastasis [47], and CD8^+^ Tex cells in metastatic tissues exhibit heightened expression of TGF-β1, IFN-γ, and ITGB1 [17]. Despite these advances, whether CD8^+^ Tex cells contribute to adenoma-to-adenocarcinoma transition has not been directly addressed. Our study demonstrates that CD8^+^ Tex cells are significantly enriched in adenoma tissues and closely associated with EPCs during malignant transformation. MIF confirmed the spatial proximity between CD8^+^ PD-1^+^ Tex cells and RUNX2^+^ EPCs, providing evidence that CD8^+^ Tex cells actively drive adenomatous EPCs transformation through direct cellular interactions.

A key finding of this study is the identification of RUNX2 as a critical transcriptional mediator linking CD8^+^ Tex cells to adenoma-to-carcinoma transformation. RUNX2 has been extensively studied as a key transcription factor in tumorigenesis and is closely associated with progression or metastasis of various cancers, including breast cancer [28], gastric cancer [29], CRC [25–27], prostate cancer [48], bladder cancer [49, 50], and cervical cancer [51]. In CRC, RUNX2 expression is significantly elevated in tumor tissues compared to adjacent normal mucosa [25, 26] and positively correlates with metastasis and poor survival [30]. Functionally, RUNX2 promotes CRC cell proliferation, migration, sphere-forming ability, and EMT [27], and transfection with RUNX2 siRNA markedly reduces these malignant properties [38]. Notably, Yingting Liu et al. demonstrated that RUNX2 plays a critical regulatory role in antitumor responses mediated by CD8^+^ T cells and CD103^+^ CD8^+^ T cells within the tumor microenvironment of human CRC [25]; however, their study did not elucidate the molecular mechanisms linking CD8^+^ T cell-derived signals to RUNX2 activation. Our study bridges this gap by establishing the TNF-α/TNFRSF1A signaling axis as the molecular link between CD8^+^ Tex cells and RUNX2 activation in EPCs during the adenoma-to-adenocarcinoma transition.

Interestingly, RUNX2 has been shown to interact with other signaling pathways. In the interferon signaling pathway, STAT1 binds to the latent form of RUNX2 in the cytoplasm, thereby inhibiting its nuclear translocation and regulating osteoblast differentiation [52]. Furthermore, RUNX2 exhibits self-regulatory properties through positive feedback loops, binding to its own promoter (particularly the E1 enhancer region) to sustain expression [53]. This self-regulatory mechanism explains why CADD522 dose-dependently reduced RUNX2 mRNA expression in HCT116 cells by disrupting the positive feedback loop. However, in the AOM/DSS model, CADD522 did not reduce the number of RUNX2^+^ EPCs, consistent with its mechanism as a transcriptional inhibitor that blocks RUNX2-DNA binding without reducing RUNX2 protein expression [45]. In the in vivo microenvironment, continuous TNF-α signaling from CD8^+^ Tex cells may sustain RUNX2 protein expression, while CADD522 could inhibit its transcriptional activity on downstream targets, thereby suppressing the adenoma-to-adenocarcinoma transition.

This study has several limitations. First, the sample size for scRNA-seq analysis was limited, and sex-stratified analyses were not performed due to the exploratory nature of this study and the small sample size per pathological stage. Second, it remains unclear whether CD8^+^ Tex cells are the sole subset driving the adenoma-to-carcinoma transition. Third, the precise mechanism by which TNF-α/TNFRSF1A signaling regulates RUNX2 activity—such as through cytoplasmic sequestration or nuclear translocation, as observed in interferon signaling [52]—requires further investigation. Finally, due to resource constraints, the spatial interaction between CD8^+^ Tex cells and EPCs was assessed solely by mIF, and future studies using advanced spatial transcriptomics techniques such as Spatial Molecular Imaging (SMI) would provide deeper mechanistic insights.

## Conclusion

In summary, this study, utilizing scRNA-seq, mIF, analyses of TCGA/GEO databases, and further in vitro and in vivo experiments, demonstrates that CD8^+^ Tex may engage TNFRSF1A on the membrane of malignant epithelial cells through their ligand TNF-α, thereby activating the transcription factor RUNX2 in COAD cells. This process promotes the transformation of malignant epithelial cells in colorectal adenomas into malignant carcinoma cells, ultimately leading to poor prognosis in COAD (Fig. 8K).

## Materials and methods

### Ethical approval and sample collection

The study protocol received approval from the Ethics Committee of the Second Affiliated Hospital of Zhejiang Chinese Medical University (No: 2025-065-IH01). All experiments were conducted strictly in accordance with the relevant guidelines and regulations. Informed consent was obtained from all subjects involved in the study. For scRNA-seq analysis, 8 samples were collected, including 1 normal tissue adjacent to the tumor, 6 mixed colorectal adenoma tissue samples, and 1 COAD tissue sample. For IHC, 16 samples were collected (4 normal tissues adjacent to the tumor, 6 colorectal adenomas, and 6 COAD samples), of which 6 representative samples (3 colorectal adenomas and 3 COAD samples) were selected for mIF. Sample size was determined based on the availability of tissue specimens and the requirements for scRNA-seq data quality.

### Sample preservation and processing for scRNA-seq

After collection, samples were preserved in tissue preservation solution (Miltenyi, Cat No. 130-100-008) for transport. Subsequently, tissues were digested into single-cell suspensions using enzymatic methods. Following the lysis of red blood cells and removal of dead cells, cell viability and quantity were assessed. Samples were then incubated with antibody solutions labeled with sample tags.

### Library construction and sequencing for scRNA-seq

Single-cell capture was performed using the BD Rhapsody system (BD, USA). Microfluidic technology was utilized to encapsulate individual cells with reaction reagents in droplets, followed by reverse transcription to construct cDNA libraries for each cell. After passing quality control, the BD Rhapsody system was used to sequence the cDNA libraries, generating a large number of reads.

### Comprehensive data acquisition and validation

ScRNA-seq datasets GSE161277 [54] and GSE201348 [55] were obtained from NCBI GEO database, along with our own data (named BD_Rhapsody). These datasets include 12 normal tissues adjacent to tumors, 37 colon adenoma tissues, and 8 COAD tissues (Table S1). Detailed patient information, including age, sex, and other clinical characteristics, is summarized in Table S2. Additionally, four transcriptomic datasets—GSE103479 [56], GSE106584 [57], GSE161158 [58], and GSE39582 [59]—were downloaded, encompassing a total of 1128 COAD samples, along with relevant clinical follow-up data. Validation was performed using the TCGA-COAD dataset and an immunotherapy cohort (IMvigor210, NCT02108652) [37].

### Preprocessing and quality control of single-cell data

Using R 4.3.3 and Seurat 4.4.0, we preprocessed the raw sequencing data from our samples and two public scRNA-seq datasets. This included quality control, removal of low-quality cells and genes, and normalization. DecontX 1.0.0 was used for the decontamination of ambient RNA [60], while DoubletFinder 2.0.3 was employed to remove doublets [61]. Cells with gene counts ≤200 or ≥8000 and a mitochondrial gene ratio ≥25% were excluded [4]. Subsequently, library size was normalized with the NormalizeData function, followed by log transformation. The FindVariableGenes function was used to identify the top 3000 highly variable genes, while Harmony 1.2.1 corrected batch effects [31]. PCA was applied for dimensionality reduction, and UMAP was utilized for data visualization. Marker genes for each cluster were determined using the FindAllMarkers function and displayed using DotPlot.

### Identification of malignant tumor cells

To assess the malignancy of EPCs, we initially utilized Cancer-Finder 1.0.0 to identify malignant cells in adenoma and COAD samples [62]. Subsequently, infercnvpy 0.4.5 (https://github.com/icbi-lab/infercnvpy) was used to detect chromosomal CNVs in these EPCs. Using immune and stromal cells as references, CNV scores were calculated for malignant epithelial cell subpopulations. EPCs with CNV scores > 0.01 were defined as highly malignant subpopulations, whereas those with CNV scores < 0.01 were considered low-malignancy subpopulations.

### Cell communication analysis

To explore the interactions between malignant EPCs and immune cells in adenoma and COAD, we isolated malignant EPCs from the samples for analysis. The CellChat 2.1.2 package was employed to analyze ligand-receptor interactions. Cell communication networks were visualized using the netAnalysis_signalingRole_scatter and netVisual_circle functions, and differential signaling pathways were illustrated with the rankNet function. To further analyze the regulatory relationship of CD8^+^ Tex cells on highly malignant EPCs, we conducted supplementary analysis using the NicheNet R package (nichenetr, v 2.1.5, https://github.com/saeyslab/nichenetr) [63].

### Pseudotime analysis

Developmental pseudotime was analyzed using Monocle2 v2.32.0. Raw counts were converted from a Seurat object to a CellDataSet object using the importCDS function. Ordering genes (*q* value < 0.01) were selected with the differentialGeneTest function. Dimensionality reduction and clustering were performed using reduceDimension, followed by trajectory inference with orderCells. Cell trajectories were visualized with plot_cell_trajectory, and gene expression heatmaps were generated using plot_pseudotime_heatmap.

### Functional enrichment analysis

KEGG and GO enrichment analyses were conducted utilizing the R packages: clusterProfiler 4.12.0, org.Hs.eg.db 3.19.1, and msigdbr 7.5.1. Gene pathways with a *P* value below 0.05 were deemed significantly enriched.

### Hematoxylin-Eosin (H-E) staining

Colon tissues were fixed in 4% paraformaldehyde solution, followed by dehydration, clearing, and paraffin embedding. The embedded tissues were sectioned into 4-5 μm thick slices using a microtome. After baking the slides for 2-3 h, H-E staining was performed using the Gemini AS automated stainer (Thermo Fisher Scientific, USA), and the slides were finally mounted with neutral balsam.

### IHC

Colon tissue paraffin blocks were sectioned, baked, and deparaffinized. Antigen retrieval was done with citrate buffer, and peroxidase was blocked using 3% hydrogen peroxide for 20 minutes. Sections were blocked with 5% BSA for 30 minutes, then incubated overnight at 4 °C with RUNX2 antibody (1:300, AFRM0095, AiFang Biobiological, China), COX-2 antibody (27308-1-AP, Proteintech, China), MMP7 antibody (3801, Cell Signaling Technology, USA), Ki67 antibody (ab16667, Abcam, UK), CD66a/b/c antibody (CEA, CPA6021, Cohesion Biosciences, China), and TNF-α antibody (60291-1-Ig, Proteintech, China), respectively. The next day, sections were incubated with HRP-conjugated secondary antibody (1:500, AFIHC003, AiFang Biobiological, China) for 60 minutes. DAB staining, hematoxylin counterstaining, dehydration, and mounting followed. IHC slides were scanned using the K-Scanner (Konfoong Biotech International Co., Ltd., Ningbo, China) and analyzed with K-Viewer software.

### MIF

MIF staining was performed on formalin-fixed paraffin-embedded (FFPE) tissue sections from COAD and CADD522-treated mice at 4 weeks (*n* = 3 per group) and 10 weeks (*n* = 3 per group), as well as human colorectal adenoma (*n* = 3) and COAD (*n* = 3) samples, using Tyramide Signal Amplification (TSA) technology. Tissue sections underwent sequential incubation with primary antibodies, subsequent secondary antibodies, and fluorophore conjugation. Multichannel imaging utilized the PANNORAMIC SCAN II Imaging System (3Dhistech, Hungary).

For multiplex immunofluorescence, tissue sections were incubated with the following primary antibodies: for human samples, RUNX2 (1:200, ab236639, Abcam, UK), Pan-CK (1:200, ZM-0069, Beijing Zhongshan Golden Bridge Biotechnology Co., Ltd., China), LAG3 (1:200, ab209236, Abcam, UK), TIM3 (1:200, ab241332, Abcam, UK), PD-1 (1:200, ZM-0381, Beijing Zhongshan Golden Bridge Biotechnology Co., Ltd., China), and CD8 (1:200, 66868-1-Ig, Proteintech, China); for mouse samples, RUNX2 (1:500, ab236639, Abcam, UK), Pan-CK (1:400, ab7753, Abcam, UK), LAG3 (1:500, HA721346, Proteintech, China), TIM3 (1:500, ab241332, Abcam, UK), PD-1 (1:400, ab214421, Abcam, UK), and CD8 (1:800, ab217344, Abcam, UK). Nuclei were counterstained with DAPI.

### Lentiviral construction and establishment of stable RUNX2 overexpression and knockdown cell lines

Lentiviral vectors for RUNX2 overexpression (RUNX2-OE), knockdown (shRUNX2), and their controls (Vector-OE and shNC) were obtained from OBiO Technology (Shanghai) Corp., Ltd. Lentiviral vectors were engineered to simultaneously express enhanced green fluorescent protein (EGFP) and confer puromycin resistance. The shRNA sequence targeting RUNX2 (shRUNX2) was TRCN0000013656: GTGGTCCTATGACCAGTCTTA. Stable cell lines were generated by infecting HCT116 and HCT15 cells with lentiviral particles at a multiplicity of infection (MOI) of 20, followed by selection with 3 μg/mL puromycin dihydrochloride (T2219, TargetMol, China) for one week and maintenance in 1 μg/mL puromycin-containing medium.

### Cell culture

NCM460 cells (C1227, WHELAB, China) and Caco-2 cells were maintained in Minimum Essential Medium (MEM) containing 20% fetal bovine serum (FBS, G8003, Wuhan Saiweier Biotechnology Co., Ltd.), 1% penicillin-streptomycin (P/S, Biosharp, China), with Caco-2 cells additionally supplemented with 1% non-essential amino acids, 1% sodium pyruvate, and 1% L-glutamine. HCT116 (STCC10803P, Wuhan Saiweier Biotechnology Co., Ltd.), HT-29 (iCell-h078, iCell, China), and HCT15 (PWE-HU127, Meilun Biotech, China) cells were cultured in McCoy’s 5A medium supplemented with 10% FBS and 1% P/S. Cell lines were authenticated via STR profiling and confirmed free of mycoplasma and chlamydia contamination.

### Cell proliferation assay

Cell proliferation was evaluated with the Cell Counting Kit-8 (CCK-8, Biosharp, China). Cells were seeded in 96-well plates at a density of 1 × 10^4^ cells per well and treated with specified reagents. After 24 h, each well received 10 μL of CCK-8 solution and was incubated at 37 °C in the dark for 2 h. Cell proliferation activity was determined by measuring absorbance at 450 nm with a microplate reader.

### Cell migration and invasion assay

Transwell chambers (8.0 μm pore size, Corning, USA) were used to evaluate cell migration and invasion capabilities. For the invasion assay, the upper chamber membrane was pre-coated with a 1:9 dilution of Matrigel (356234, BD Biosciences, USA). HCT116 cells and HCT15 cells (1×10^5^/well) suspended in serum-free medium were placed in the upper chamber, with McCoy’s 5A containing 20% FBS as a chemoattractant in the lower chamber. After 24 h incubation (37 °C, 5% CO_2_), non-migrated or invaded cells on the upper surface were carefully removed using cotton swabs. Subsequently, migrated/invaded cells were fixed in 4% paraformaldehyde for 20 min, stained with 0.1% crystal violet for 15 min, and quantified using ImageJ software.

### QRT-PCR

Total RNA was extracted from colon tissue using Trizol (Invitrogen, Waltham, MA, USA). RNA concentration was assessed using a NanoDrop One spectrophotometer (Thermo Fisher Scientific, USA), and the measured concentration was 500 ng/μL. For reverse transcription, 500 ng of total RNA was used with the PrimeScript^TM^ RT Master Mix (Takara, China). QRT-PCR was performed using TB Green® Premix Ex Taq^TM^ (Takara, China). The expression of target genes in tissues was normalized using GAPDH. The 2^-ΔΔCt^ method [64] was employed to calculate the relative expression levels of each target gene. Refer to Table S3 for the primer sequences.

### WB

Colon tissue was lysed with RIPA buffer (BL504A, Biosharp, China) containing PMSF (BL1426A, Biosharp, China). Protein was quantified using a BCA kit (BL521A, Biosharp, China). Samples underwent electrophoresis, membrane transfer, blocking (1 h), and incubation with primary RUNX2 antibody (1:1000, ab236639, Abcam, UK, overnight at 4 °C) and secondary antibodies (1:5000, 1 h). Bands were visualized using the LICOR Odyssey DLx system (Gene Company Limited, Hong Kong) and quantified with Image J.

### Establishment of a mouse colorectal adenoma-carcinoma model using AOM/DSS

A mouse colorectal adenoma/carcinoma model was established using the AOM/DSS method. Male C57BL/6 mice (6-8 weeks old) were randomly assigned to either the COAD group or the RUNX2 inhibitor CADD522 treatment group (*n* = 15 per group). On the first day of the experiment, all mice received an intraperitoneal injection of azoxymethane (AOM, 10 mg/kg). Six days later, the mice underwent three cycles of 2% DSS treatment in drinking water (7 days of DSS administration followed by 14 days of regular drinking water per cycle). The CADD522 group was administered CADD522 (20 mg/kg, intraperitoneally, every three days) starting after the AOM injection and continuing until the end of the experiment, while the COAD group received an equal volume of solvent control. At 4, 7, and 10 weeks, 5 mice per group were euthanized, and colon tissues were collected for further analysis.

### Establishment of a mouse CRC xenograft model

Male BALB/c nude mice (4-6 weeks old, nu/nu) were sourced from Hangzhou Qizhen Experimental Animal Technology Co., Ltd. The mice were randomly divided into four groups (*n* = 5 per group). For xenograft experiments, HCT116 cells stably transfected with RUNX2 overexpression vector (RUNX2-OE), RUNX2 shRNA (shRUNX2), or their corresponding control vectors (Vector-OE and shNC, respectively) were used. Under isoflurane anesthesia, mice were subcutaneously injected with 0.1 mL cell suspension (5 × 10^6^ viable cells) in the right axillary region. Subcutaneous tumor measurements were performed every three days. Tumor volume was determined using the formula *V* = 1/2 × *L* × *W*^2^, where L is the longest, and W is the shortest perpendicular tumor diameter. At day 25 post-implantation, mice were humanely euthanized, and the xenograft tumors were harvested and weighed. All animal procedures were approved by the Institutional Animal Care and Use Committee of Zhejiang Chinese Medical University (IACUC-20241028-15). All experiments were conducted strictly in accordance with the relevant guidelines and regulations.

### Statistical analysis

Statistical and graphical analyses were performed using GraphPad Prism 10.0 software. Data are presented as mean ± standard deviation (SD). For comparisons between two groups, an independent two-sample t-test (two-tailed) was used. For comparisons among three or more groups, one-way analysis of variance (ANOVA) was performed, followed by Tukey’s post hoc test for multiple comparisons. Statistical significance was defined as a *p* value < 0.05, with specific thresholds denoted as ^*^P < 0.05, ^**^P < 0.01, ^***^P < 0.001, ^****^P < 0.0001. Each experiment was repeated independently with similar results.

## Supplementary information

Supplement1-Table S1

Supplement1-Table S2

Supplement2

Original western blots

## Data Availability

The datasets supporting the conclusions of this study are available in the GEO database (https://www.ncbi.nlm.nih.gov/geo/) and TCGA database (https://portal.gdc.cancer.gov/). Other data supporting the findings of this study are available from the corresponding author upon reasonable request.
