# Supplementary material for: RUNX2 drives adenoma-to-carcinoma transition in colon cancer
Source: Cell Death Dis. 2026 Apr 29;17(1):575. doi: 10.1038/s41419-026-08801-2 (PMC13272649; doi:10.1038/s41419-026-08801-2)
Supplement: Supplementary file 1 — Supplement1-Table S1 [file 41419_2026_8801_MOESM1_ESM.pdf]

		Donor Info	Sample Info		Pathology
**Source**	**Sample**	**Donor**	**GrossPathology**	**Location**	**PolypType**
GSE201348	A001-C-119	A001	Polyp	Transverse	Tubular Adenoma
GSE201348	A001-C-203	A001	Polyp	Ascending	Tubular Adenoma
GSE201348	A001-C-207	A001	Polyp	Ascending	Tubular Adenoma
GSE201348	A002-C-010	A002	Polyp	Descending	Tubular Adenoma
GSE201348	A002-C-016	A002	Polyp	Descending	Tubular Adenoma
GSE201348	A002-C-021	A002	Polyp	Descending	Tubular Adenoma
GSE201348	A002-C-106	A002	Polyp	Transverse	Tubular Adenoma
GSE201348	A002-C-114	A002	Polyp	Transverse	Tubular Adenoma
GSE201348	A002-C-116	A002	Polyp	Transverse	Tubular Adenoma
GSE201348	A002-C-201	A002	Polyp	Ascending	Tubular Adenoma
GSE201348	A002-C-203	A002	Polyp	Ascending	Tubular Adenoma
GSE201348	A002-C-204	A002	Polyp	Ascending	Tubular Adenoma
GSE201348	A002-C-205	A002	Polyp	Ascending	Tubular Adenoma
GSE201348	A014-C-001	A014	Polyp	Descending	Tubular Adenoma
GSE201348	A014-C-008	A014	Polyp	Rectum	Tubular Adenoma
GSE201348	A014-C-040	A014	Polyp	Descending	Tubular Adenoma
GSE201348	A014-C-043	A014	Polyp	Descending	Tubular Adenoma
GSE201348	A014-C-101	A014	Polyp	Transverse	Tubular Adenoma
GSE201348	A015-C-002	A015	Polyp	Descending	Tubular Adenoma
GSE201348	A015-C-104	A015	Polyp	Transverse	Tubular Adenoma
GSE201348	A015-C-202	A015	Polyp	Ascending	Tubular Adenoma
GSE201348	A015-C-203	A015	Polyp	Ascending	Tubular Adenoma
GSE201348	A015-C-204	A015	Polyp	Ascending	Tubular Adenoma
GSE201348	F007	F	Polyp	Ascending	Tubular Adenoma
GSE201348	F034	F	Polyp	Transverse	Tubular Adenoma
GSE201348	F072B	F	Polyp	Descending	Tubular Adenoma
GSE201348	F091	F	Polyp	Rectum	Tubular Adenoma
GSE201348	A001-C-007	A001	Adenocarcinoma	Descending	Adenocarcinoma
GSE201348	CRC-1-8810	CRC1	Adenocarcinoma		
GSE201348	CRC-2-15564	CRC2	Adenocarcinoma		
GSE201348	CRC-3-11773	CRC3	Adenocarcinoma		
GSE201348	A001-C-023	A001	Unaffected	Descending	Normal
GSE201348	A001-C-223	A001	Unaffected	Ascending	Normal
GSE201348	A002-C-121	A002	Unaffected	Transverse	Normal
GSE201348	A002-C-212	A002	Unaffected	Ascending	Normal
GSE201348	A014-C-052	A014	Unaffected	Rectum	Normal
GSE201348	A014-C-054	A014	Unaffected	Descending	Normal
GSE201348	A014-C-114	A014	Unaffected	Transverse	Normal
GSE201348	A015-C-008	A015	Unaffected	Rectum	Normal
GSE201348	B001-A-301	B001	Normal	Sigmoid	Normal
GSE201348	B001-A-401	B001	Normal	Transverse	
GSE201348	B001-A-406	B001	Normal	Descending	Normal
GSE201348	B001-A-501	B001	Normal	Ascending	Normal
GSE201348	B004-A-004	B004	Normal	Sigmoid	
GSE201348	B004-A-008	B004	Normal	Descending	
GSE201348	B004-A-104	B004	Normal	Transverse	
GSE201348	B004-A-204	B004	Normal	Ascending	
GSE161277	GSM4904235	Patient1	Adenoma		
GSE161277	GSM4904236	Patient1	Carcinoma		
GSE161277	GSM4904237	Patient1	Normal		
GSE161277	GSM4904238	Patient2	Adenoma		
GSE161277	GSM4904238	Patient2	Adenoma		
GSE161277	GSM4904239	Patient2	Carcinoma		
GSE161277	GSM4904240	Patient2	Normal		
GSE161277	GSM4904242	Patient3	Adenoma		
GSE161277	GSM4904243	Patient3	Adenoma		
GSE161277	GSM4904246	Patient3	Normal		
This study	P001	P001	Adenoma	Ascending	Tubular Adenoma
This study	P002	P002	Adenoma	Transverse	Tubular Adenoma
This study	P003	P003	Adenoma	Descending	Tubular Adenoma
This study	P004	P004	Adenoma	Transverse	Tubular Adenoma
This study	P005	P005	Adenoma	Sigmoid	Tubular Adenoma
This study	P006	P006	Adenoma	Sigmoid	Tubular Adenoma
This study	P007-1	P007	Normal		Normal
This study	P007-2	P007	Adenocarcinoma		Adenocarcinoma

Samples P001-P007 were collected from our clinical cohort. The original data for other samples in this study were obtained from GSE161277 (https://www.ncbi.nlm.nih.gov/gds/?term=GSE161277) and GSE201348 (https://www.ncbi.nlm.nih.gov/gds/?term=GSE201348). From GSE161277, we selected samples that met the requirements of this study, and from GSE201348, we selected samples with consistent Gross Pathology and Microscopic Pathology results that fulfilled the study criteria, as shown in the table above.
