# Supplementary material for: RUNX2 drives adenoma-to-carcinoma transition in colon cancer
Source: Cell Death Dis. 2026 Apr 29;17(1):575. doi: 10.1038/s41419-026-08801-2 (PMC13272649; doi:10.1038/s41419-026-08801-2)
Supplement: Supplementary file 2 — Supplement1-Table S2 [file 41419_2026_8801_MOESM2_ESM.pdf]

Source	Patient ID	Disease Status	Age	Sex	Cancer Status	tissue processed
This study	P001	Adenoma	61	F	N/A	polyp
This study	P002	Adenoma	55	F	N/A	polyp
This study	P003	Adenoma	56	M	N/A	polyp
This study	P004	Adenoma	70	M	N/A	polyp
This study	P005	Adenoma	71	M	N/A	polyp
This study	P006	Adenoma	64	F	N/A	polyp
This study	P007	Adenocarcinoma	62	M	Adenocarcinoma	normal/cancer
GSE201348	A001	FAP	47	M	Adenocarcinoma	normal/polyp/cancer
GSE201348	A002	FAP	22	F	N/A	normal/polyp
GSE201348	A014	FAP	22	F	N/A	normal/polyp
GSE201348	A015	FAP	35	F	Adenocarcinoma	normal/polyp
GSE201348	B001	Healthy	67	F	N/A	normal
GSE201348	B004	Healthy	78	M	N/A	normal
GSE201348	F	FAP	53	M	N/A	polyp
GSE201348	CRC1 8810	Adenocarcinoma	61	M	Adenocarcinoma	cancer
GSE201348	CRC2 15564	Adenocarcinoma	66	F	Adenocarcinoma, medullary type	cancer
GSE201348	CRC3 11773	Adenocarcinoma	55	M	Adenocarcinoma	cancer
GSE161277	Patient1	Adenocarcinoma	61	M	Adenocarcinoma	normal/polyp/cancer
GSE161277	Patient2	Adenocarcinoma	64	M	Adenocarcinoma	normal/polyp/cancer
GSE161277	Patient3	Adenocarcinoma	53	F	Adenocarcinoma	normal/polyp/cancer

Samples P001-P007 were collected from our clinical cohort. The original data for other samples in this study were obtained from GSE161277 (https://www.ncbi.nlm.nih.gov/gds/?term=GSE161277) and GSE201348 (https://www.ncbi.nlm.nih.gov/gds/?term=GSE201348). From GSE161277, we selected samples that met the requirements of this study, and from GSE201348, we selected samples with consistent Gross Pathology and Microscopic Pathology results that fulfilled the study criteria, as shown in the table above.
