# Supplementary material for: RUNX2 drives adenoma-to-carcinoma transition in colon cancer
Source: Cell Death Dis. 2026 Apr 29;17(1):575. doi: 10.1038/s41419-026-08801-2 (PMC13272649; doi:10.1038/s41419-026-08801-2)
Supplement: Supplementary file 3 — Supplement2 [file 41419_2026_8801_MOESM3_ESM.docx]

### Supplement

**Table**

**Table S3 Sequences of primers used for qRT-PCR**

| **Sequences of primers used for qRT-PCR** | | | |
| --- | --- | --- | --- |
| Name |  | Sequence (5' to 3') | Supplier |
| CEBPB-H | Forward | CTTCAGCCCGTACCTGGAG | Sangon Biotech (Shanghai) Co., Ltd. |
|  | Reverse | GGAGAGGAAGTCGTGGTGC |  |
| RUNX2-H | Forward | TCAACGATCTGAGATTTGTGGG |  |
|  | Reverse | GGGGAGGATTTGTGAAGACGG |  |
| ANXA2-H | Forward | TGCCTTCGCCTACCAGAGAA |  |
|  | Reverse | GCCCAAAATCACCGTCTCC |  |
| SERPINE2-H | Forward | TGGTGATGAGATACGGCGTAA |  |
|  | Reverse | GTTAGCCACTGTCACAATGTCTT |  |
| LEF1-H | Forward | AGAACACCCCGATGACGGA |  |
|  | Reverse | GGCATCATTATGTACCCGGAAT |  |
| MAT2A-H | Forward | ACCAGAAAGTGGTTCGTGAAG |  |
|  | Reverse | CAAGGCTACCAGCACGTTACA |  |
| NOTCH1-H | Forward | TGGACCAGATTGGGGAGTTC |  |
|  | Reverse | GCACACTCGTCTGTGTTGAC |  |
| STAT1-H | Forward | CGGCTGAATTTCGGCACCT |  |
|  | Reverse | CAGTAACGATGAGAGGACCCT |  |
| MLLT6-H | Forward | ACAAAGACGGGGCATTGAAGA |  |
|  | Reverse | AGCACGTTGGCAAATTGCAC |  |
| KI67-H | Forward | ACGCCTGGTTACTATCAAAAGG |  |
|  | Reverse | CAGACCCATTTACTTGTGTTGGA |  |
| p53-H | Forward | CAGCACATGACGGAGGTTGT |  |
|  | Reverse | TCATCCAAATACTCCACACGC |  |
| MMP7-H | Forward | ATGTGGAGTGCCAGATGTTGC |  |
|  | Reverse | AGCAGTTCCCCATACAACTTTC |  |
| COX2-H | Forward | TAAGTGCGATTGTACCCGGAC |  |
|  | Reverse | TTTGTAGCCATAGTCAGCATTGT |  |
| CEA-H | Forward | TCTTGGCTGATTGATGGGAAC |  |
|  | Reverse | CACTGGCTGAGTTATTGGCCT |  |
| KI67-M | Forward | ATCATTGACCGCTCCTTTAGGT |  |
|  | Reverse | GCTCGCCTTGATGGTTCCT |  |
| MMP7-M | Forward | TCGCAAGGAGAGATCATGGAG |  |
|  | Reverse | CTGCGTCCTCACCATCAGTC |  |
| COX2-M | Forward | TGAGCAACTATTCCAAACCAGC |  |
|  | Reverse | GCACGTAGTCTTCGATCACTATC |  |
| CEA-M | Forward | ATTTCACGGGGCAAGCATACA |  |
|  | Reverse | GTCACCCTCCACGGGATTG |  |

**Figure**

**Figure S1**

**
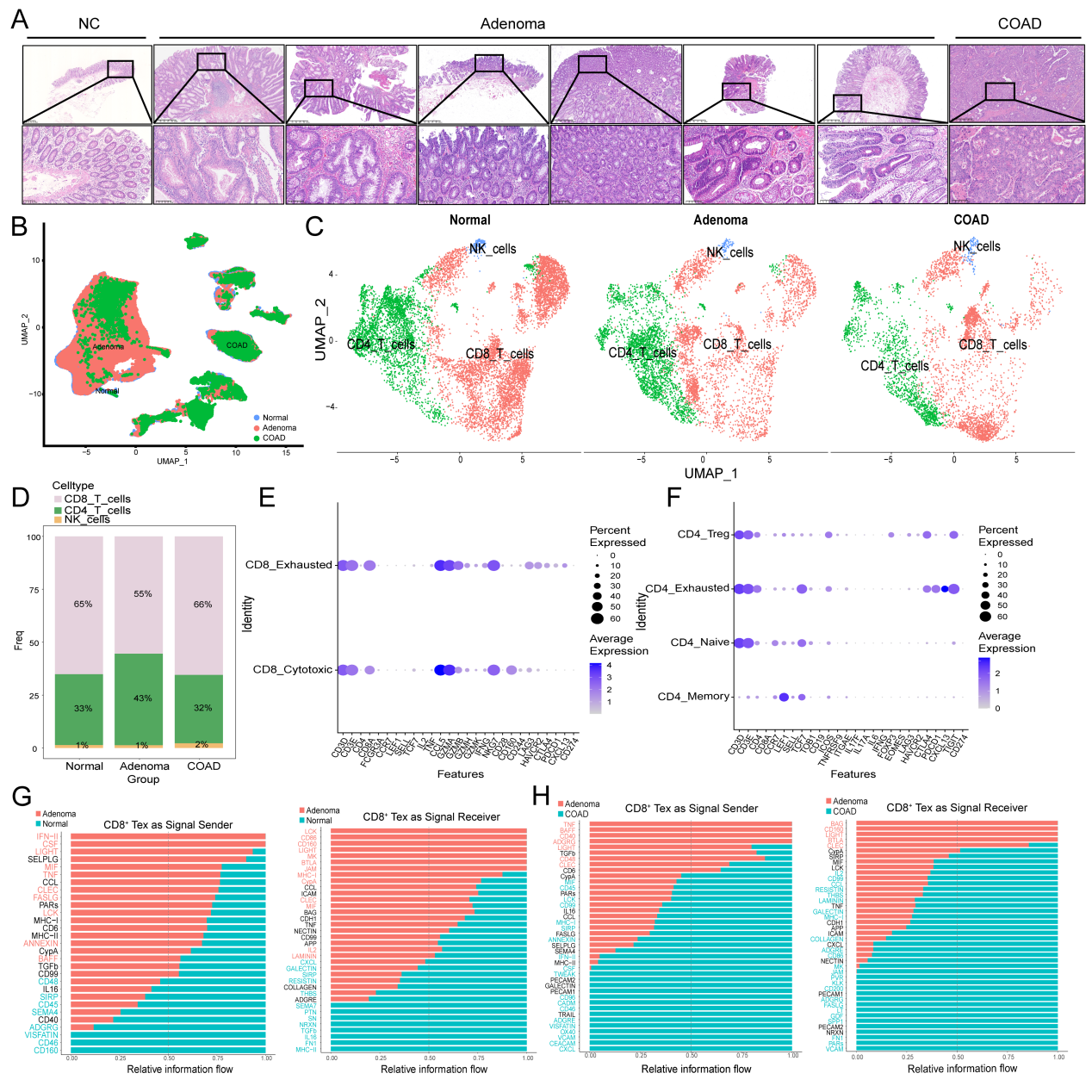
**

Figure S1. (A) Representative HE staining images of colon tissues from patients used for scRNA-seq analysis. (B) UMAP plot of integrated single-cell data colored by tissue type. (C) UMAP plots showing the distribution of NK cells, CD8^+^ T cells, and CD4^+^ T cells in normal, adenoma, and COAD tissues. (D) Bar plot depicting the proportions of major T cell subtypes across groups. (E) Dot plot showing marker gene expression for CD8^+^ T cell subclusters. (F) Dot plot showing marker gene expression for CD4^+^ T cell subclusters. (G)Bar plot depicting the relative information flow of signaling pathways in CD8^+^ Tex as signal sender or receiver in normal and adenoma tissues. (H) Bar plot depicting the relative pathway information flow of CD8^+^ Tex as signal sender or receiver in COAD and adenoma tissues.

**Figure S2**

**
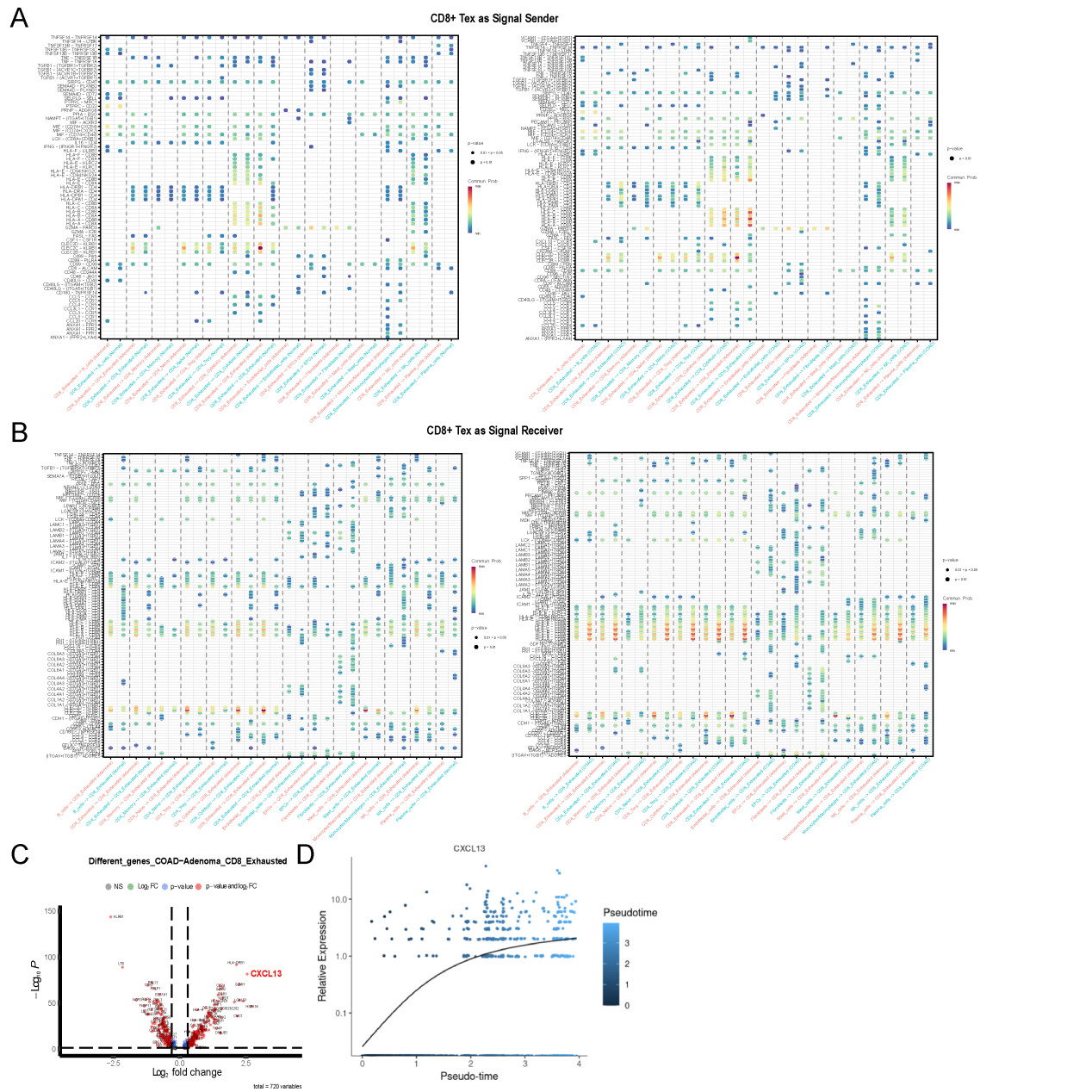
**

Figure S2. (A) CD8^+^ Tex as signal sender (ligand-expressing cell): Left: Comparison between adjacent normal tissue (NC) and adenoma. Right: Comparison between adenoma and COAD. (B) CD8^+^ Tex as signal receiver (receptor-expressing cell): Left: Comparison between NC and adenoma. Right: Comparison between adenoma and COAD. The color of each dot represents the communication probability, and the dot size indicates statistical significance (*P* value). (C) Volcano plot of differentially expressed genes between COAD- and adenoma-derived CD8^+^ Tex. (D) Dynamic expression of CXCL13 along the pseudotime trajectory.

**Figure S3**

**
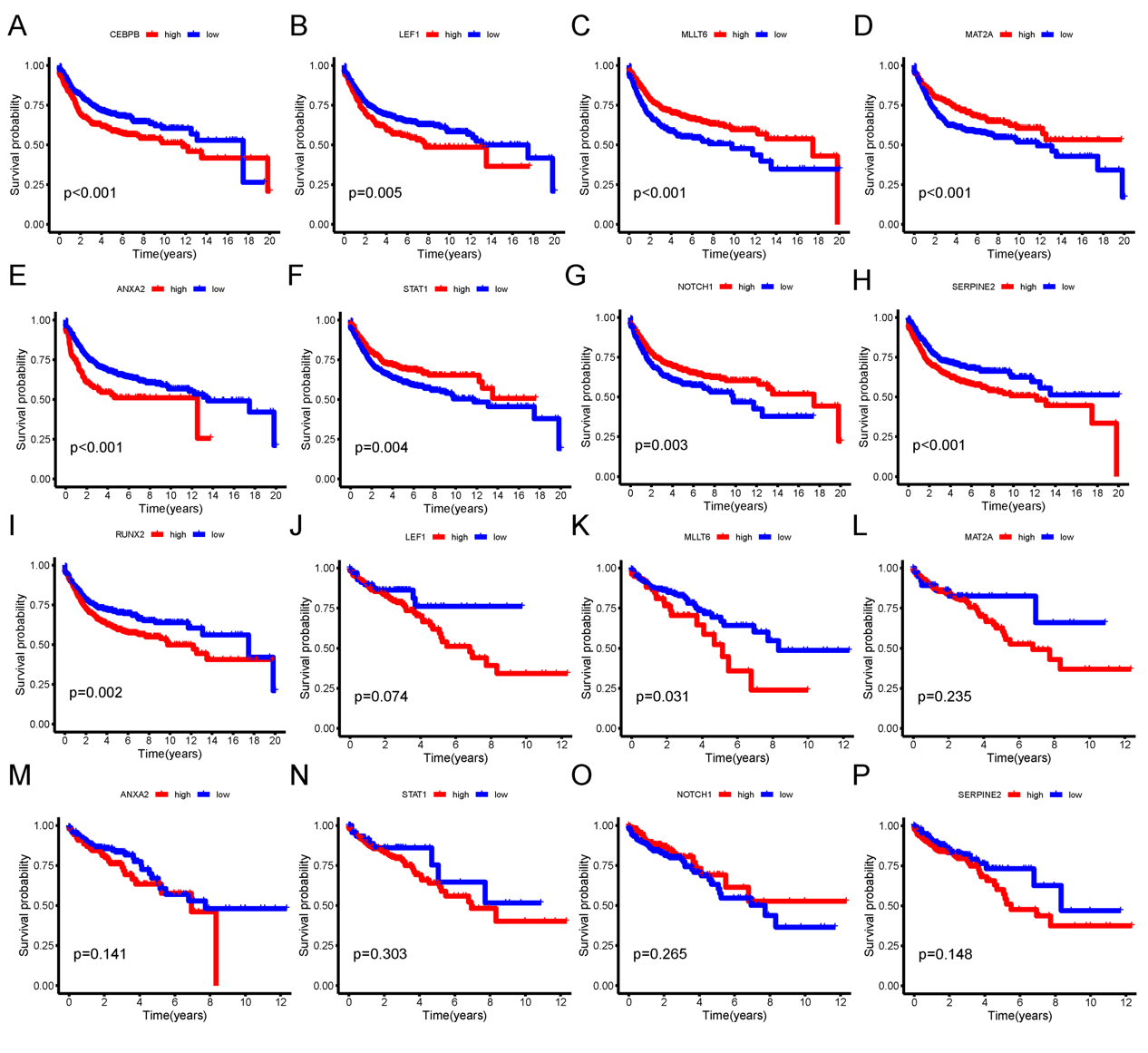
**

Figure S3. Survival analysis of model genes. (A-I) Survival analysis of model genes CEBPB, LEF1, MLLT6, MAT2A, ANXA2, STAT1, NOTCH1, SERPINE2, and RUNX2 in the GEO database. (J-P) Validation of survival analysis for model genes LEF1, MLLT6, MAT2A, ANXA2, STAT1, NOTCH1, and SERPINE2 in the TCGA database. In each plot, the red curve represents the high-expression group and the blue curve represents the low-expression group. The x-axis indicates follow-up time (years), and the y-axis indicates survival probability. The *P* value in each panel reflects the statistical significance of the survival difference between the two groups.

**Figure S4**

**
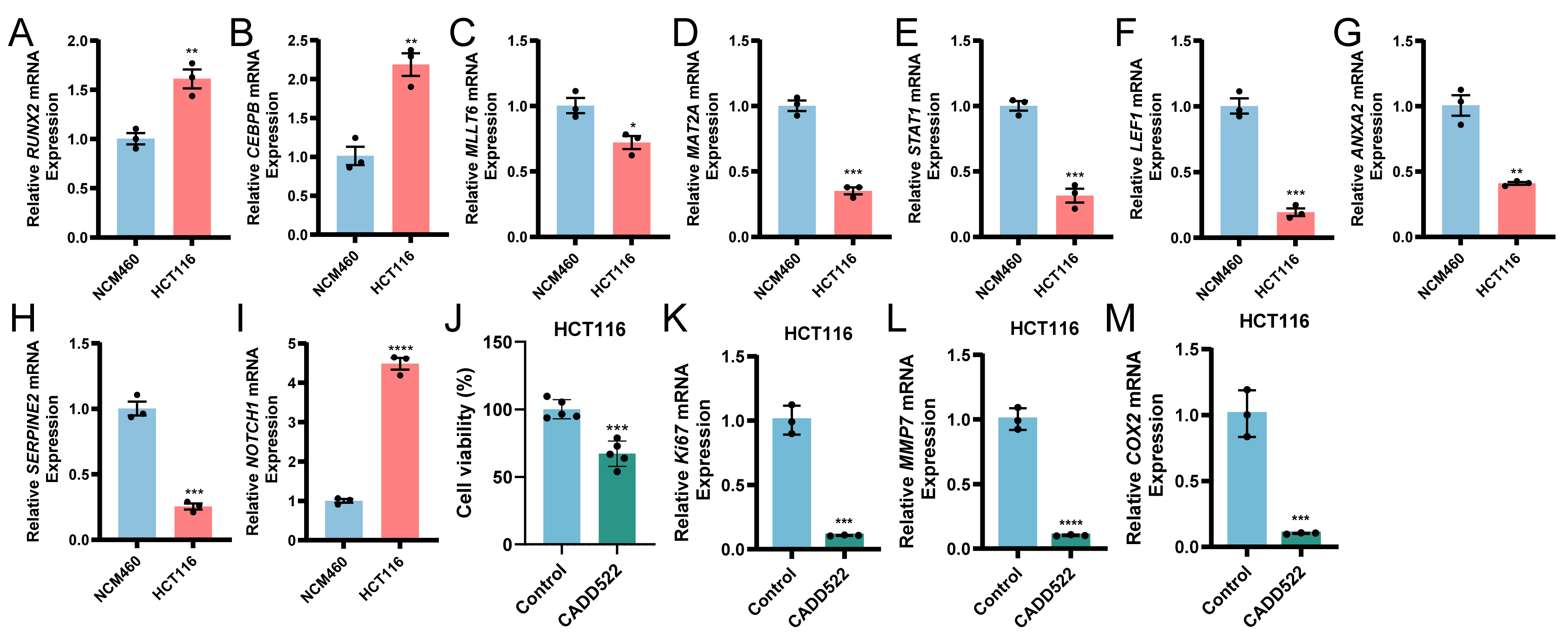
**

Figure S4. (A-I) Relative mRNA expression levels of *RUNX2*, *CEBPB*, *MLLT6*, *MAT2A*, *STAT1*, *LEF1*, *ANXA2*, *SERPINE2*, and *NOTCH1* in NCM460 and HCT116 cells. (J) Effect of CADD522 on the viability of HCT116 cells. (K-M) Relative mRNA expression levels of *Ki67*, *MMP7*, and *COX-2* in COAD cells following CADD522 treatment in vitro.

**Figure S5**


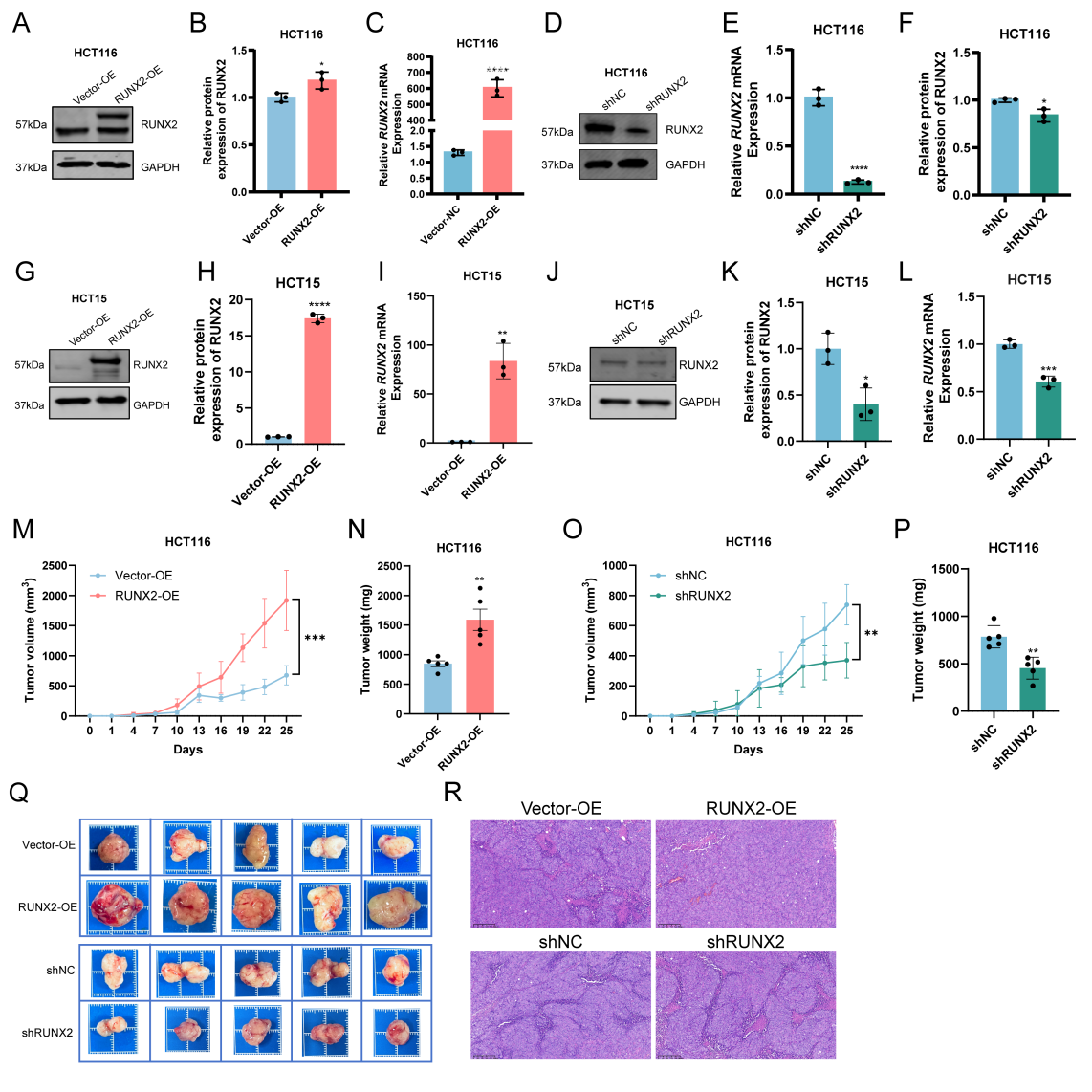


Figure S5. (A-C) Overexpression of RUNX2 (RUNX2-OE) in HCT116 cells confirmed by WB and qRT-PCR analyses. (D-F) Knockdown of RUNX2 (shRUNX2) in HCT116 cells confirmed by WB and qRT-PCR analyses. (G-I) Overexpression of RUNX2 (RUNX2-OE) in HCT15 cells confirmed by WB and qRT-PCR analyses. (J-L) Knockdown of RUNX2 (shRUNX2) in HCT15 cells confirmed by WB and qRT-PCR analyses. (M-N) RUNX2 overexpression promotes tumor growth in HCT116 cells, as demonstrated by increased tumor volume and weight in the RUNX2-OE group compared to the Vector-OE group. (O-P) RUNX2 knockdown inhibits tumor growth in HCT116 cells, as demonstrated by reduced tumor volume and weight in the shRUNX2 group compared to the shNC group. (Q) Representative images of tumors from the Vector-OE, RUNX2-OE, shNC and shRUNX2 groups. (R) HE staining showing histological differences between tumors from the Vector-OE, RUNX2-OE, shNC, and shRUNX2 groups.

**Figure S6**


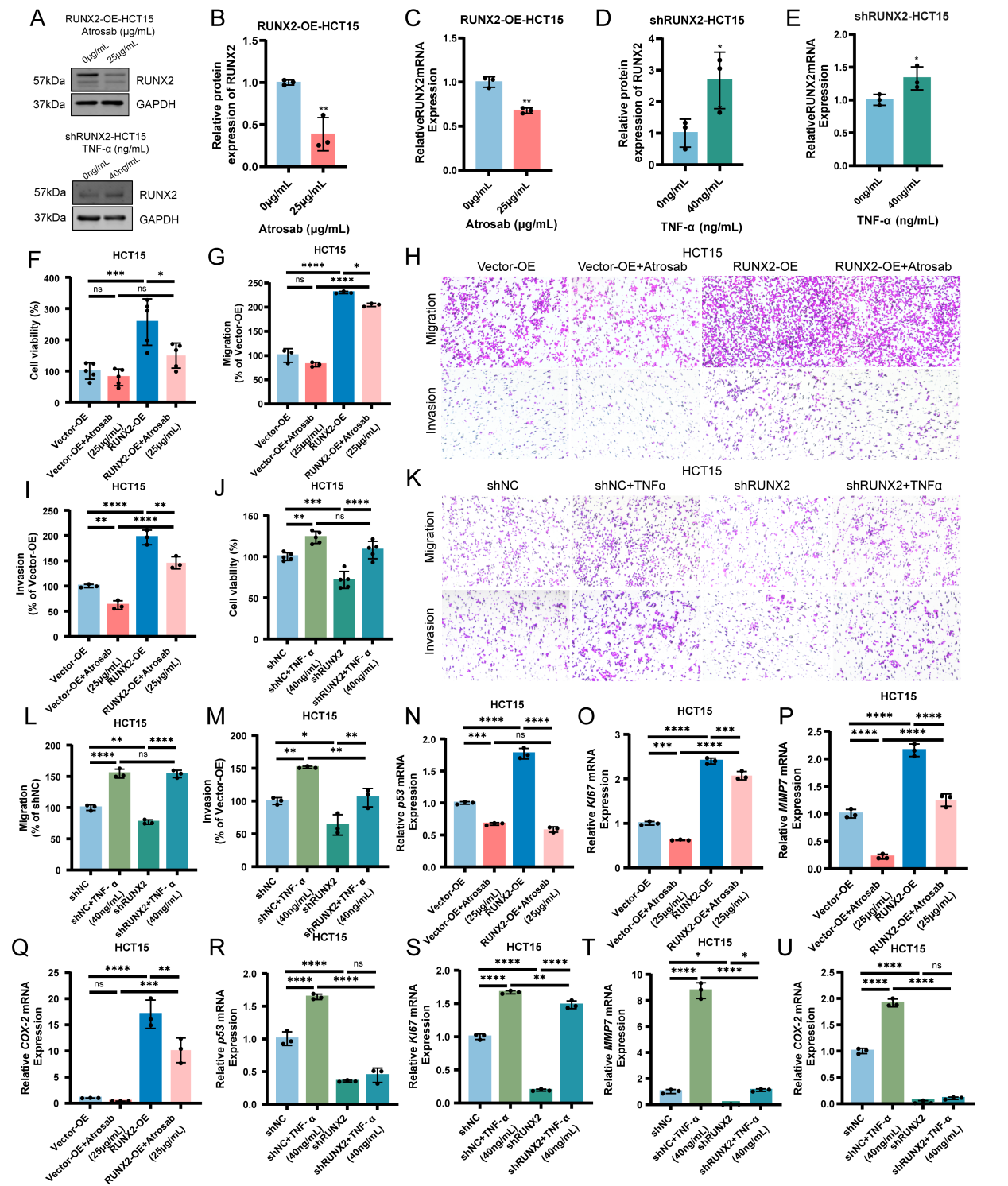


Figure S6. (A) RUNX2 protein expression in RUNX2-overexpressing HCT15 cells (RUNX2-OE-HCT15) after treatment with Atrosab (25μg/mL) and in RUNX2-knockdown HCT15 cells (shRUNX2-HCT15) after treatment with TNF-α (40ng/mL). (B, C) Relative RUNX2 protein and mRNA expression in RUNX2-overexpressing HCT15 cells after treatment with Atrosab. (D, E) Relative RUNX2 protein and mRNA expression in RUNX2-knockdown HCT15 cells after treatment with TNF-α. (F-I) Effects of the TNFRSF1A inhibitor Atrosab on the proliferation, migration, and invasion abilities of control (Vector-OE) and RUNX2-OE-HCT15 cells. (J-M) Effects of the TNFRSF1A agonist TNF-α on the proliferation, migration, and invasion abilities of shNC and shRUNX2-HCT15 cells. (N-U) mRNA expression levels of genes associated with adenoma-to-adenocarcinoma transition (*p53*, *Ki67*, *MMP7*, *COX-2*) in each group of HCT15 cells.

**Figure S7**

**
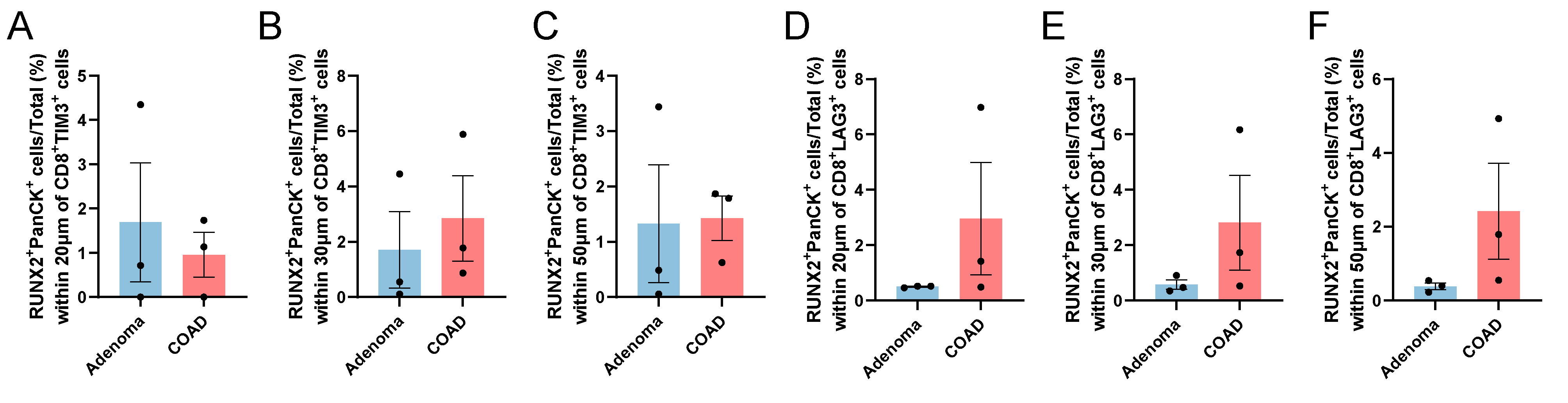
**

Figure S7. (A-C) Spatial distance analysis showing the proportion of RUNX2^+^ EPCs within 20 μm (A), 30 μm (B), and 50 μm (C) of CD8^+^ TIM3^+^ Tex in each group. (D-F) Spatial distance analysis showing the proportion of RUNX2^+^ EPCs within 20 μm (D), 30 μm (E), and 50 μm (F) of CD8^+^ LAG3^+^ Tex in each group.
