# Supplementary figures and images for: RUNX2 drives adenoma-to-carcinoma transition in colon cancer
Source: Cell Death Dis. 2026 Apr 29;17(1):575. doi: 10.1038/s41419-026-08801-2 (PMC13272649; doi:10.1038/s41419-026-08801-2)

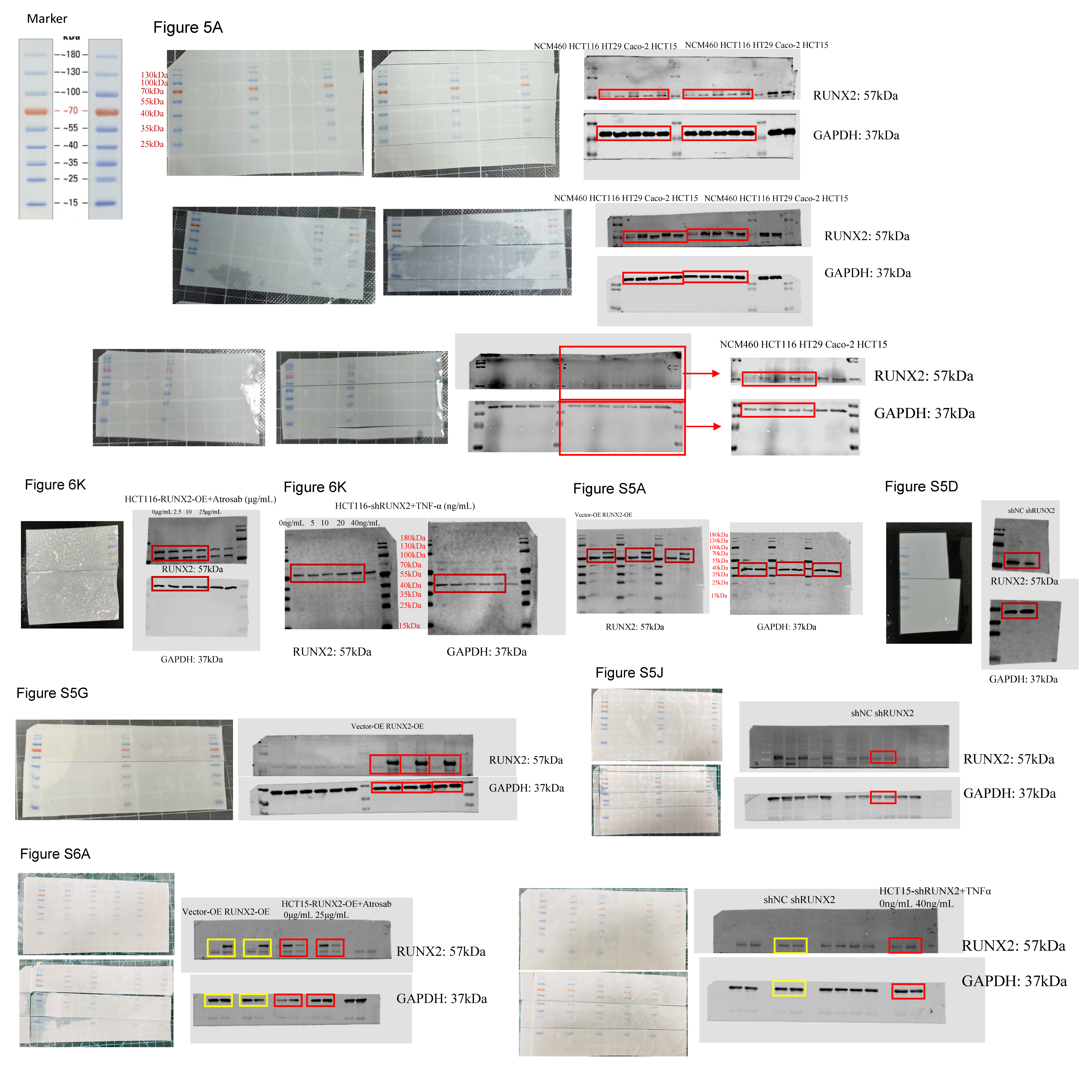

Supplement: Supplementary file 4 — Original western blots [file 41419_2026_8801_MOESM4_ESM.tif]
